# Comprehensive Modelling of the *Neurospora* Circadian Clock and Its Temperature Compensation

**DOI:** 10.1371/journal.pcbi.1002437

**Published:** 2012-03-29

**Authors:** Yu-Yao Tseng, Suzanne M. Hunt, Christian Heintzen, Susan K. Crosthwaite, Jean-Marc Schwartz

**Affiliations:** 1 Faculty of Life Sciences, University of Manchester, Manchester, United Kingdom; 2 Manchester Interdisciplinary Biocentre, Faculty of Life Sciences, University of Manchester, Manchester, United Kingdom; University of Virginia, United States of America

## Abstract

Circadian clocks provide an internal measure of external time allowing organisms to anticipate and exploit predictable daily changes in the environment. Rhythms driven by circadian clocks have a temperature compensated periodicity of approximately 24 hours that persists in constant conditions and can be reset by environmental time cues. Computational modelling has aided our understanding of the molecular mechanisms of circadian clocks, nevertheless it remains a major challenge to integrate the large number of clock components and their interactions into a single, comprehensive model that is able to account for the full breadth of clock phenotypes. Here we present a comprehensive dynamic model of the *Neurospora crassa* circadian clock that incorporates its key components and their transcriptional and post-transcriptional regulation. The model accounts for a wide range of clock characteristics including: a periodicity of 21.6 hours, persistent oscillation in constant conditions, arrhythmicity in constant light, resetting by brief light pulses, and entrainment to full photoperiods. Crucial components influencing the period and amplitude of oscillations were identified by control analysis. Furthermore, simulations enabled us to propose a mechanism for temperature compensation, which is achieved by simultaneously increasing the translation of *frq* RNA and decreasing the nuclear import of FRQ protein.

## Author Summary

Circadian clocks are internal timekeepers that integrate signals from the environment and orchestrate cellular events to occur at the most favourable time of day. Circadian clocks in animals, plants, fungi and bacteria have similar characteristic properties and molecular architecture. They have a periodicity of approximately 24 hours, persist in constant conditions and can be reset by environmental time cues such as light and temperature. Another essential property, whose molecular basis is poorly understood, is that the period is temperature compensated i.e. it remains the same over a range of temperatures. Computational modelling has become a valuable tool to predict and understand the underlying mechanisms of such complex molecular systems, but existing clock models are often restricted in the scope of molecular reactions they cover and in the breadth of conditions they are able to reproduce. We therefore built a comprehensive model of the circadian clock of the fungus *Neurospora crassa*, which encompasses existing knowledge of the biochemistry of the *Neurospora* clock. We validated this model against a wide range of experimental phenotypes and then used the model to investigate possible molecular explanations of temperature compensation. Our simulations suggest that temperature compensation of period is achieved by changing the abundance and cellular localisation of a key clock protein.

## Introduction

Circadian clocks are essential endogenous timekeepers that are present in most organisms. They impose temporal order on inter- and intracellular events [1], regulating the cell cycle [2], cell physiology [3] and behaviour [4]. Circadian rhythmicity emerges from a network of positive and negative feedback regulation acting on clock genes and clock proteins [5], [6]. Ubiquitous characteristics of circadian clocks include: a periodicity of approximately 24 h, the ability to be entrained to the external rhythmic environment, and temperature compensation of period [7]. Synchronisation of circadian clocks to local time allows organisms to anticipate and prepare for cyclical changes in their environment. Of the defining clock characteristics, temperature compensation is the least understood.

At the molecular level, a common characteristic of circadian clocks is that there is usually an underlying transcriptional and translational feedback loop [reviewed in 8]. Positive and negative elements regulate the expression of clock genes and clock-controlled genes. For example, KaiA in *Synechococcus* [reviewed in 9], CLK and CYC in *Drosophila* [reviewed in 10], and Clock and Bmal1 (Mop3) in mammals [reviewed in 11] are positive elements. These molecules promote the expression of the clock and clock-controlled genes. On the other hand, negative elements, such as KaiC in *Synechococcus* [reviewed in 9], PERIOD and TIMELESS in *Drosophila* [reviewed in 10], and Cry1, Cry2, Per1 and Per2 in mammals [reviewed in 11], repress the action of positive elements. The rhythmic regulation results in periodic expression of the clock genes and clock-controlled genes. Consequently, rhythmic changes in metabolism and behaviour can be observed [reviewed in 12].

**Figure 1:**
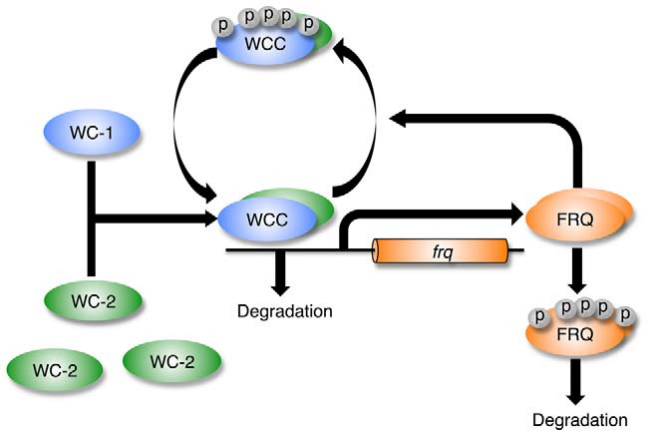
Simplified representation of the *Neurospora* circadian clock. Transcription factors WHITE COLLAR-1 (WC-1) and WHITE COLLAR-2 (WC-2) form a heterodimeric WHITE COLLAR COMPLEX (WCC). Early in the subjective night, the hypophosphorylated form of WCC (hypoWCC) activates the transcription of the *frequency* (*frq*) gene. Once hypoWCC activates transcription, it is degraded. The FREQUENCY protein (FRQ) accumulates, peaking around midday, and is progressively phosphorylated. Hyperphosphorylated FRQ is ubiquitinated and degraded by the proteosome. FRQ promotes phosphorylation of WCC by recruiting kinases, and phosphorylated WCC (hyperWCC) is inactive thus leading to decreased transcription of *frq* and consequently negative regulation of FRQ. Phosphorylated WCC is more stable than its hypophosphorylated form, thus the increase in FRQ level leads to a rise in overall WCC level.

The *Neurospora crassa* clock is based on molecular feedback loops that in constant conditions generate a 22 hour period (Figure 1). Components include the *frequency* (*frq*), *white collar-1* (*wc-1*) and *white collar-2* (*wc-2*) genes. In the positive loop, the White Collar Complex (WCC), a heterodimer of WC-1 and WC-2, activates the transcription of *frq*. The product of the *frq* gene, the FREQUENCY (FRQ) protein, transcriptionally and post-transcriptionally promotes the accumulation of WC-2 and WC-1, respectively [13], [14]. In a negative feedback loop, FRQ recruits kinases, such as casein kinase-1a (CK-1a), and facilitates WCC phosphorylation [15]. The phosphorylation of WCC results in WCC inactivation and thus interferes with the binding of WCC to the *frq* promoter [16]. Moreover, the WCC represses *wc-2* transcription via up-regulation of a putative repressor [17].

Interaction between environmental factors, such as light, and the *Neurospora* clock components is well-studied [18]. WC-1 and VIVID (VVD) proteins are known blue light receptors [19], [20], [21]. Light stimulates the formation of a large photoactivated WCC (laWCC), containing more than one WC-1 molecule. laWCC is transformed from the heterodimeric dark WCC, and even in the presence of FRQ is a strong transcriptional activator of *frq*, as well as *vvd* and a number of clock-controlled genes [reviewed in 5]. The blue light receptor VVD acts as a repressor of light-induced responses [22]. Evidence suggests that VVD competes with laWCC for the homodimerisation of laWCC and this results in the dissociation of laWCC and a concomitant decrease in its ability to activate transcription [23], [24], [25].

Given the large number of components and processes involved in the circadian clockwork it becomes ever more difficult to interpret its functioning and response to environmental factors by intuition and reasoning alone. A rigorous, quantitative model that embeds our knowledge of the circadian network should make it possible to test the consequences of experimental perturbations on the system, and reveal components and mechanisms underlying clock characteristics. Such a model would allow predictions to be made regarding the behaviour of the clockwork under a wide variety of conditions. Quantitative models of the *Neurospora* circadian clock have been built previously [26], [27], [28], [29], [30], [31]. Leloup's minimal *Neurospora* clock model concentrates on *frq* gene expression which is regulated by the concentration of FRQ protein using the Hill equation. The model was the first to successfully simulate both a period of 21.5 hours in constant darkness and entrainment of the oscillator to a 24 hour light-dark cycle. Ruoff *et al.* developed a model, based on a Goodwin-type oscillator, that introduces a switch mechanism to activate and repress *frq* transcription [30]. Temperature-regulated degradation of wild type and mutant forms of FRQ was modelled by introducing the Arrhenius equation, resulting in the expected expression of *frq* mRNA and FRQ at 21°C and 28°C. This work and subsequent experiments have shown that wild type FRQ degradation is not significantly affected over this range of temperatures [32]. François' model considers an interaction between FRQ and *wc-1* RNA, and the inactivation of WCC through binding with FRQ homodimers [29]. Subsequent modelling by Hong *et al.* has provided insight into the possible mechanism of FRQ action in the nucleus indicating that a one-to-one molar ratio of FRQ and WCC is not necessary for FRQ to repress WCC activity [31].

As shown by the above examples, quantitative modelling has made valuable contributions to our understanding of circadian clock mechanism in *Neurospora*. To date however, no model is able to describe the full range of observed clock phenotypes. Because the *Neurospora* circadian clockwork consists of several interlocking feedback loops, a comprehensive model is expected to shed light on circadian clock properties and mechanisms underlying its response to environmental factors, in particular temperature compensation. Our model incorporates the majority of the known clock components and the mechanisms through which they interact, and successfully accounts for a wide range of clock characteristics including: a periodicity of approximately 24 hours, persistent oscillation in conditions of constant darkness and temperature, arrhythmicity in constant light, resetting of the clock by brief pulses of light, photoadaptation, and entrainment to full photoperiods. Relative levels of clock gene transcripts and clock proteins mimic the experimentally derived values in constant darkness, after light pulses and in light/dark conditions. Control analysis carried out on the model and comparisons between model simulations and experimental data allow us to propose a mechanism underlying temperature compensation.

## Results

### The *Neurospora crassa* circadian clock model

**Figure 2:**
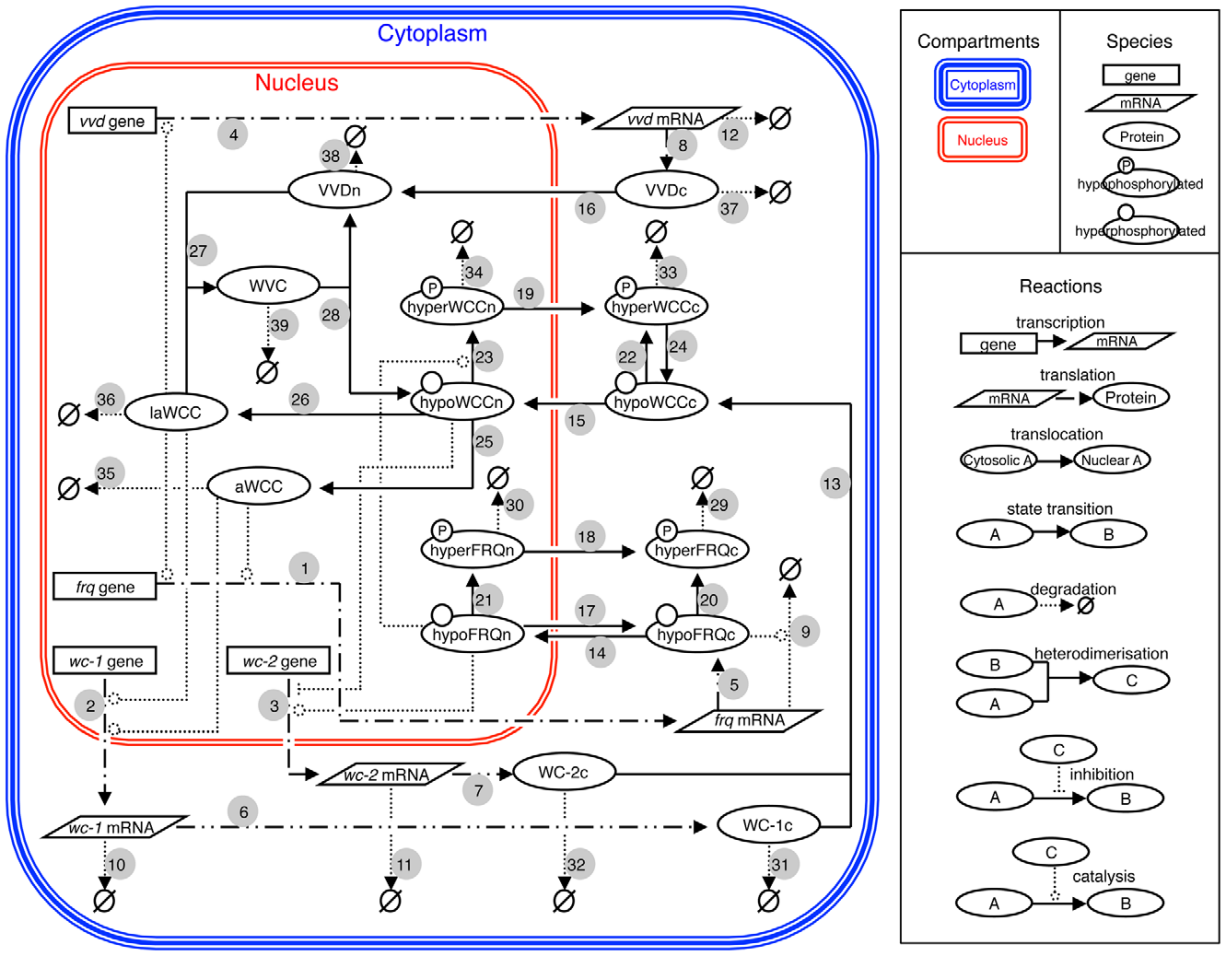
The *Neurospora* circadian clock model. The symbol representations of compartments, species and reactions are shown in the right hand panel. Individual pathways are numbered starting with the transcription of the *frq* gene. *frq* = *frequency*, *wc-1* = *white collar-1*, *wc-2* = *white collar-2*, *vvd* = *vivid*, hypoFRQc = cytosolic hypophosphorylated FREQUENCY (FRQ) protein, hypoFRQn = nuclear hypophosphorylated FRQ, hyperFRQc = cytosolic hyperphosphorylated FRQ, hyperFRQn = nuclear hyperphosphorylated FRQ, WC-1c = cytosolic WHITE COLLAR-1 (WC-1) protein, WC-2c = cytosolic WHITE COLLAR-2 (WC-2) protein, hypoWCCc = cytosolic hypophosphorylated WHITE COLLAR COMPLEX (WCC), hypoWCCn = nuclear hypophosphorylated WCC, hyperWCCc = cytosolic hyperphosphorylated WCC, hyperWCCn = nuclear hyperphosphorylated WCC, aWCC = activated WCC, laWCC = light activated WCC, VVDc = cytosolic VIVID (VVD) protein, VVDn = nuclear VVD, WVC = WCC-VVD complex.

Our circadian clock model was constructed through a mechanistic approach (Figure 2). The model is based on a compilation of published and new (this paper) experimental data and incorporates facets of previously described *Neurospora* clock models [28], [29], [31]. The model centres on the genetic interlocking positive and negative feedback loops created by the interactions of the *frq*, *wc-1* and *wc-2* genes [reviewed in 5], [33]. The *frq*, *wc-1* and *wc-2* genes are transcribed into *frq*, *wc-1* and *wc-2* mRNA (step 1–3), respectively, and translated into hypophosphorylated cytosolic FRQ (hypoFRQc), WC-1 (WC1c) and WC-2 (WC2c) protein (step 5–7). Steps 9–11 are degradation reactions of *frq* mRNA, *wc-1* mRNA, and *wc-2* mRNA.

Once translated, cytoplasmic WC-1 (WC1c) and WC-2 (WC2c) bind to each other to form the hypophosphorylated cytosolic WHITE-COLLAR complex (hypoWCCc) (step 13) which translocates into the nucleus (step 15) where a small fraction of hypoWCCn is activated (activated WCC) (step 25) [14]. Activated WCC promotes the transcription of *frq* and *wc-1* genes (steps 1 and 2) [34], [35] and as a consequence is degraded [14] (step 35). Hypophosphorylated cytosolic WCC (hypoWCCc) and nuclear WCC (hypoWCCn) can be phosphorylated in the cytoplasm and in the nucleus (step 22 and 23). Hyperphosphorylated nuclear WCC (hyperWCCn) is translocated out of the nucleus (step 19) to the cytoplasm where it can be dephosphorylated (step 24) [14]. Once translated, FRQ forms a homodimer that interacts with the FRQ-interacting helicase FRH [36]; in our model this complex is represented by FRQ. Hypophosphorylated nuclear FRQ (hypoFRQn) facilitates the phosphorylation of hypoWCCn [16] (step 23) and clearance of WCC from the nucleus [14]. Thus, FRQ negatively regulates its own expression and positively regulates the accumulation of WCC.

Steps 31–35 are degradation reactions of WC-1c, WC-2c, hyperWCCc, hyperWCCn and activated WCC, respectively. hypoFRQ shuttles into (step 14) and out of (step 17) the nucleus [37] and is progressively phosphorylated in both the cytoplasm (step 20) and the nucleus (step 21) [reviewed in 38]. Hyperphosphorylated nuclear FRQ (hyperFRQn) is translocated out of the nucleus (step 18) and accumulates in the cytoplasm [37]. Hyperphosphorylated FRQ can be degraded in the cytoplasm (step 29) and nucleus (step 30). In addition, *wc-2* transcription is promoted by hypophosphorylated nuclear FRQ (hypoFRQn) [17] and *wc-2* transcription is repressed by hypophosphorylated nuclear WCC (hypoWCCn) [17] (step 3).

The main blue-light response components, i.e. WCC and VVD are incorporated in the model. WC-1 is a photoreceptor and transcription factor which enhances the transcription of light responsive genes [20], [39], [40]. VIVID functions as a repressor of the light response [22], [23], [24], [25]. WCC can be activated by light (step 26) and promotes transcription of *frq* (step 1), *wc-1* (step 2) and *vvd* (step 4). The expression of *vvd* is clock-controlled, but after the first day in constant darkness its expression is much reduced (no expression can be detected by northern blot). Activated WCC induces *vvd* in the light (step 4) [22]. Once translated (step 8) VIVID (VVD) is transported into the nucleus (step 16), where it interferes with the function of light activated WCC (laWCC) by competing with laWCC subunits and preventing their homodimerisation [23], [25]. Steps 27 and 28 describe the formation and the disassociation of the WCC-VVD complex (WVC). Steps 12, 36–39 are degradation reactions of light activated WCC (laWCC), *vvd* mRNA, cytosolic VVD (VVDc), nuclear VVD (VVDn) and the WCC-VVD complex (WVC). The full set of kinetic equations and parameter values used in the model are presented in Tables S1 and S2.

### Clock simulation in constant darkness

**Figure 3:**
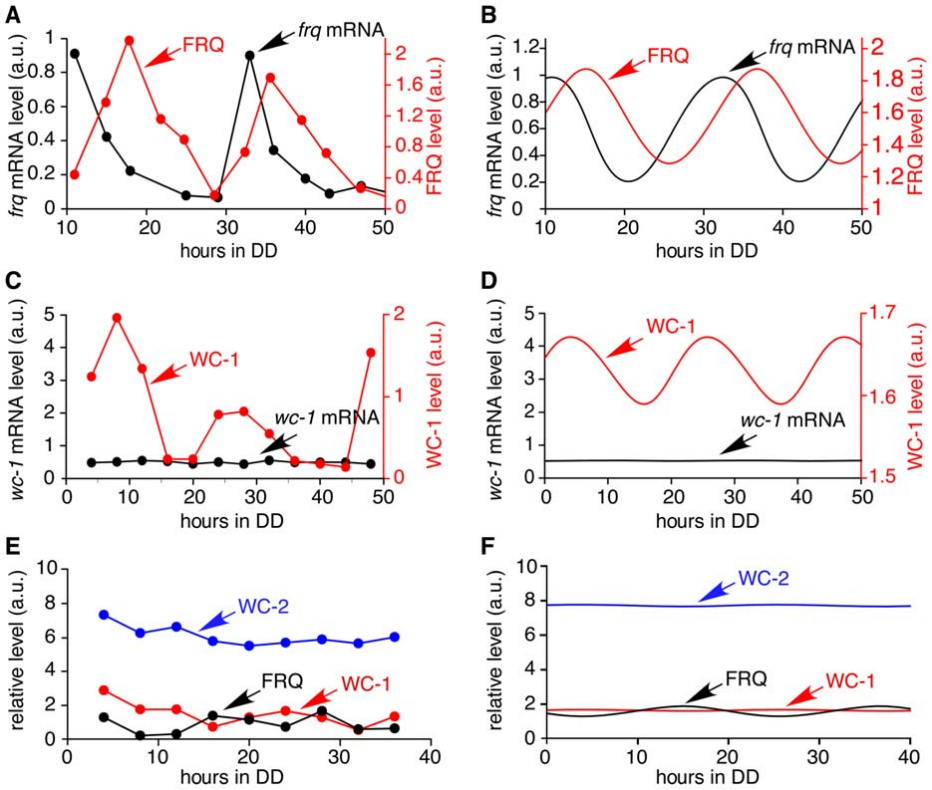
Continuous dark simulations. (A) Experimental data showing the oscillation of *frq* mRNA and FRQ protein levels [41]. The period length is approximately 22 hours, and FRQ (red line) peaks 3–7 hours after *frq* mRNA (black line). (B) Simulated results showing the oscillation of *frq* mRNA and FRQ protein levels has a period of 21.6 hours, and FRQ peaks 4.4 hours after *frq* mRNA. Simulation begins 10 hours after a light to dark transfer, 10 data points per hour. (C) Experimental data showing *wc-1* mRNA and WC-1 protein levels [42]. The level of *wc-1* mRNA is nearly constant. WC-1 protein expression oscillates. (D) Simulated results. *wc-1* mRNA (black line) level is constant and WC-1 oscillates. Simulation begins at the light to dark transfer, 10 data points per hour. (E) The level of WC-2 protein is 5–30 times higher than the average level of FRQ and WC-1 protein [43]. (F) In the model WC-2 protein is 10 times higher than the average level of FRQ and WC-1 proteins. Simulation begins at the light to dark transfer, 10 data points per hour are plotted.

A comparison of model simulations and experimental data from the literature is shown in Figure 3. Rhythmic expression of *frq* mRNA and protein is seen in continuous darkness (DD) with *frq* mRNA peaking at CT 0–4 with a period of 21.6 h and FRQ protein peaking 3–7 hours later (Figure 3A) [41]. A plot of the simulated oscillations of *frq* mRNA and FRQ protein (Figure 3B) shows that the period (21.6 h) and amplitude of *frq* mRNA and FRQ are similar to experimental results. The delay between peak levels of *frq* and FRQ is 4.3 hours, which lies inside the range of experimental results. The simulated behaviours of these core clock components are in agreement with the experimental data from Garceau *et al.* (1997). While *wc-1* transcription is not rhythmic, WC-1 oscillates with peak levels around 18–20 hours after the light to dark transition, 8 hours after peak levels of FRQ (Figure 3C [42] and 3D (simulated results)). Figure 3E and 3F show that there is a good match between experimentally determined [43] and simulated levels of clock proteins.

### Model robustness to parameter perturbation

To evaluate the robustness of the model we determined the range of each parameter value within which rhythmicity was maintained, and in which the period and amplitude of the rhythm remained within experimentally defined limits. The oscillation of *frq* RNA was used as the reference for these tests. To determine how much each parameter value can change while still generating oscillations, each parameter was increased and decreased until *frq* RNA oscillation was lost (Table S3). Our results show that 8 parameters are restricted to a fairly small range of values, 12 parameters can be decreased to zero, 8 parameters can be increased to infinity and 4 parameters can take any value without losing oscillations.

We determined how much each parameter value can change while remaining compatible with the experimental wild-type *Neurospora* clock period, taking a reference value of 21.6 hours and an experimental standard error of 0.6 hours (estimated from race tube experiments). Each parameter was increased and decreased until the period was increased or decreased outside the range of 21.6±0.6 hours (Table S4). Some parameters are highly sensitive since the oscillation is lost before the target increase or decrease of period can be reached. Other parameters can be modified in a certain range of values while remaining compatible with the experimental range of observed periodicity.

A similar approach was taken to determine how much each parameter value can change while remaining compatible with the experimental amplitude of *frq* RNA oscillations, taking an uncertainty of ±5%. Each parameter was increased and decreased until the amplitude of *frq* RNA was increased or decreased by 5% of its original value (Table S5). Most parameters are highly constrained by the amplitude; 25 parameters cannot be changed by more than 10% without changing the amplitude by more than 5%. The remaining 7 parameters can take a large range of values without affecting the amplitude strongly.

Taken together, these tests show that with the exception of *ka_wc2* (FRQ-induced transcription of *wc-2*), *kout_hyperFRQn* (translocation of phosphorylated FRQ out of the nucleus), *kd_hyperFRQc* and *kd_hyperFRQn* (degradation of phosphorylated FRQ in the cytoplasm and nucleus, respectively), most parameters are highly constrained. The low sensitivity of FRQ-induced transcription of *wc-2* is expected because the basal transcription level of *wc-2* is high in comparison. The low sensitivity of hyperphosphorylated FRQ related parameters is due to the fact that hyperphosphorylated FRQ has no function in the model. The high sensitivity of other parameters suggests that if the clock properties are to be maintained over a wide range of environmental conditions, complex adjustments of multiple parameters are necessary.

**Figure 4:**
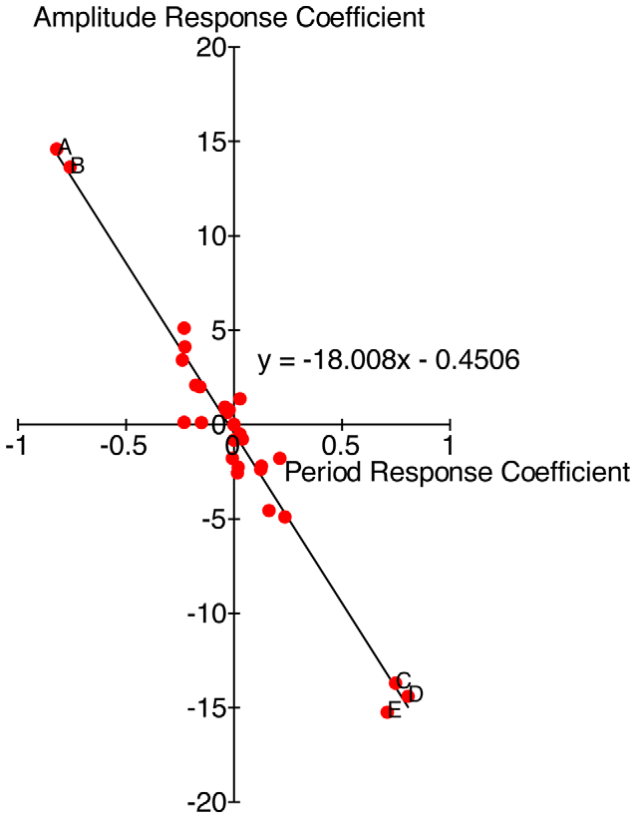
Distribution of parameters based on the value of their period and amplitude response coefficients. The trend line indicates that period and amplitude response coefficients are negatively correlated. A = *wc-1* translation, B = rate of basal transcription of *wc-1*, C = Michaelis constant of *frq* transcription, D = degradation of *wc-1* mRNA, E = degradation of activated WCC.

Response coefficient analysis indicates that the period of the oscillator and the amplitude of *frq* mRNA is most sensitive to the Michaelis constant of *frq* transcription, the rate of basal transcription of *wc-1*, *wc-1* translation, degradation of *wc-1* mRNA, and degradation of activated WCC (Figures S1 and S2). A 10% decrease of *wc-1* basal transcription or translation rate, or a 10% increase of the Michaelis constant of *frq* transcription, degradation of activated WCC or *wc-1* mRNA, all result in a substantially dampened oscillator (Figure 4) with a longer period (e.g. decreasing basal *wc-1* transcription rate by 10% results in a period of 25.8 hours). A negative correlation is observed between period and amplitude response coefficients of most clock components, showing that an increase in the amplitude of oscillations would result in faster regulation through feedback loops, if not compensated (Figure 4).

### Phenotypes of *Neurospora* with a mutant form WC-2 or an inducible copy of *wc-1*

**Figure 5:**
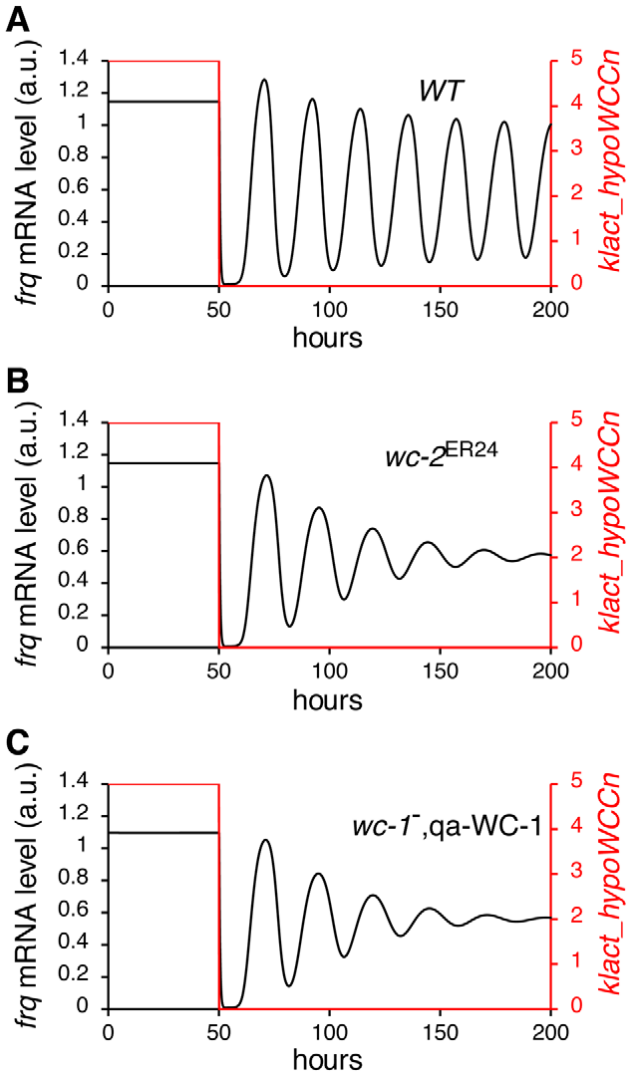
Reproduction of *wc-2* ^ER24^ mutant and *wc-1* ^−^, qa-WC-1 behaviour. (A) Simulated results showing levels of *frq* RNA and light-activated WCC_n_ before and after a light to dark transfer. 10 data points/h are plotted. When the light (red line) is turned off after 50 h, *frq* mRNA levels oscillate. (B) Simulated *frq* mRNA behaviour of the *wc-2* ^ER24^ mutant and (C) the *wc-1* ^−^, qa-WC-1 strain. In (B) and (C) *frq* mRNA oscillates in the dark but the oscillation dampens with time.

From the response coefficient analysis we found that changes in the basal transcription rate of *wc-1* and the dissociation constant of WCC binding to the *frq* promoter (*K_frq*) have a large effect on the period and amplitude of the oscillator (Figure 5). Phenotypes resulting from altered transcription of *wc-1* and binding of the WCC are seen in mutant and engineered strains of *Neurospora*. For example *wc-2^ER24^* is a *Neurospora* mutant that displays reduced WCC binding at the *frq* promoter due to a mutation at a conserved position in its Zn finger DNA-binding domain [44]. *wc-2^ER24^* has a long period (∼29.7 hours) and becomes arrhythmic after 3–4 circadian days at 25°C [44]. In *wc-2^ER24^*, *frq* mRNA levels are lower and peak levels of *frq* are delayed compared to wild-type. In addition, the oscillation of FRQ protein dampens with time [44]. Figure 5A shows the simulation of light to dark transfer of wild-type *Neurospora*. When we increase the dissociation constant of WCC binding to the *frq* promoter *K_frq* by 10% our model successfully reproduces the characteristics of the *wc-2^ER24^* mutant (Figure 5B).

Experimentally, the basal rate of *wc-1* transcription has been altered by introducing an inducible copy of the *wc-1* gene (qa-WC-1). In the qa-WC-1 strain the WC-1 ORF is fused to the quinic acid-inducible promoter (*qa-2*) [13] and transcription of *wc-1* is controlled by the concentration of quinic acid (QA) in the medium. Conidiation in a qa-WC-1 expressing strain is arrhythmic when the concentration of QA is low (1×10^−7^ M), but shows sustained rhythmicity at 1×10^−4^ M QA. At 1×10^−5^ M QA, conidial rhythms rapidly dampen after 3–4 cycles. In the model, *k_wc1* is the rate of basal transcription of *wc-1*. Similar to *K_frq*, *k_wc1* is also sensitive to the period and the amplitude of oscillation. Decreasing *k_wc1* by 10% results in a substantially dampened oscillation, successfully reproducing the behaviour of qa-WC-1 banding at 1×10^−5^ M QA (Figure 5C).

### Simulation of light response phenotypes

**Figure 6:**
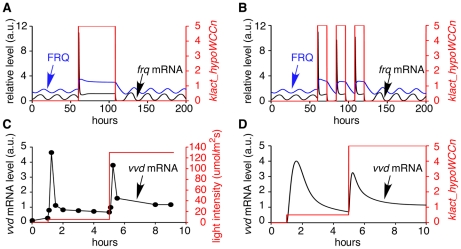
Simulated results of light resetting, entrainment by light/dark (LD) cycles, and photoadaptation. (A) The simulation consists of 60 h dark, 48 h constant light, 92 h constant dark. 10 data points per hour are plotted. The red line indicates light intensity. After the dark to light transfer, *frq* mRNA and FRQ protein increase and the oscillation is lost. Rhythmic expression begins without delay on return to darkness. (B) The simulation began with continuous dark for 60 hours, followed by three cycles of 12 h light: 12 h dark, before transfer to constant dark. The simulated clock can be entrained to 24 h with 12 h: 12 h light: dark cycles. (C) Experimental data shows the molecular behaviour of photoadaptation [25]. Light intensity (red line) of 5 mol·m^−2^·s^−1^ for 4 hours and 130 mol·m^−2^·s^−1^ for 5 h. *vvd* mRNA level (black line) is rapidly and transiently induced to high levels immediately after exposure to light. (D) The behaviour of *vvd* mRNA photoadaptation is reproduced in the model. A second increase in light intensity results in a rapid increase of *vvd* RNA. The levels soon fall but remain at a higher level compared to levels at the lower light intensity. 100 data points per hour were plotted.

To simulate the response of the *Neurospora* clock to light we incorporated light-activated WCC (laWCC) into the model (step 26 in Figure 2). laWCC strongly activates transcription of *frq*, *wc-1* and *vvd* (step 1, 2 and 4). Photoadaptation occurs as laWCC function is quickly diminished by the formation of the laWCC VVD complex (WVC) (step 27). After interacting with VVD, the laWCC dissociates and returns to its dark state (hypoWCCn) (step 28) [25]. Light results in a rapid but transient increase of *frq* mRNA and FRQ protein [45], [46], after which levels of *frq* and FRQ quickly drop to a level close to their peak levels in darkness (Figure 6A). Figure 6B shows a simulation of light/dark cycles. The period of cycling *frq* RNA and protein is entrained to 24 hours in the light dark cycles, and on return to continuous dark the clock exhibits its free running periodicity of 21.6 h. Although the phase of the clock is delayed by approximately two hours compared to the experimentally determined phase, the magnitude of the delay can be increased or decreased by changing the kinetics of FRQ-dependent *frq* RNA degradation [47] and of VVD's interaction with light-activated WCC. These data are consistent with results that show that VVD plays a role in setting the phase of the clock at the light dark transition [22], [48], [49]. In agreement with experimental data [25] (Figure 6C), on exposure to light of increasing intensity, after a transient increase of *frq* RNA and protein, photo-adaptation occurs and the photo-adapted steady state of gene expression depends on light intensity (Figure 6D).

**Figure 7:**
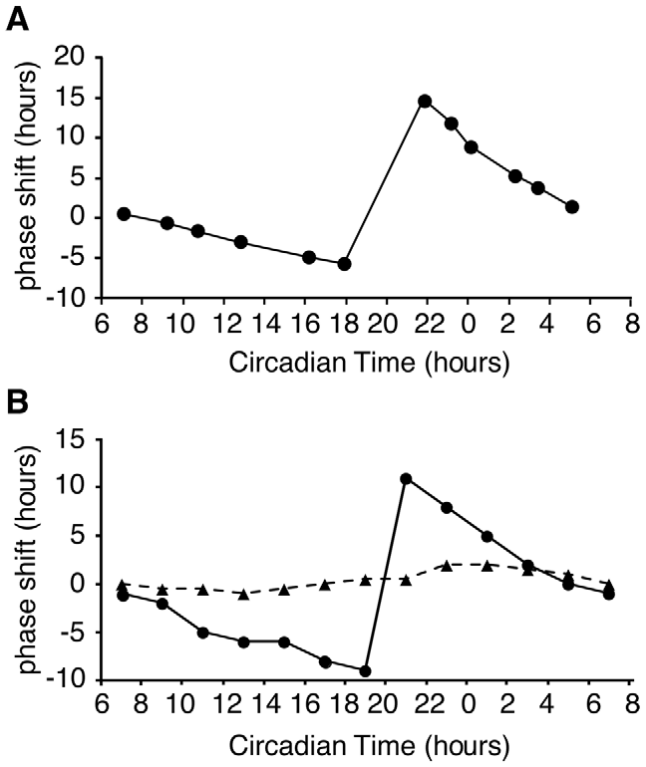
Phase response curves. (A) Plot of experimental data showing light-induced phase shifts [45]. Light pulses given during the late subjective night and early morning result in large phase shifts. (B) Light-induced phase shifts are reproduced in the model. 0.1 h light pulses (solid line) cause larger phase shifts than 0.01 h light pulses (dashed line). Large phase shifts occurred during the late subjective night and early morning. 20 and 200 data points/h were plotted for simulated 0.1 and 0.01 h light pulse respectively.

A universal characteristic of circadian clocks is their phase response to light. Light exposure during the late subjective night advances their phase of oscillation. In contrast, light exposure late in the subjective day results in phase delays and light exposure during the day results in little or no change in clock time (Figure 7A) [50], [51]. To examine the effect of light pulses on our model clock, the system was pulsed with light (duration 0.1 or 0.01 h) at 2 hour intervals covering one circadian day. In the model, large phase shifts are induced during the (late) subjective day and early morning (Figure 7B), which is in agreement with the published literature [45] (Figure 7A). Moreover, simulating the phase shift behaviour with different amounts of light reproduces the dependency of phase shift magnitude on the amount of light [52] (Figure 7B, dotted line).

### Temperature compensation

In principle temperature compensation of period may be achieved because clock component activity is unaffected by temperature, or because temperature affects the activity of more than one clock component such that the net effect is no change in period [53], [54], [55], [56]. Whilst some reaction rates are seemingly temperature-insensitive, temperature-dependent changes in the binding affinity, activity or conformation of the proteins involved has usually occurred [32], [57], [58]. For example, though the degradation rate of FRQ is temperature compensated, as the temperature rises different sites on the protein are phosphorylated by CK2. It is likely that these sites become available for phosphorylation due to a temperature-induced change in FRQ conformation or/and temperature-induced changes in the binding activity of CK2. As a result of this phosphorylation the degradation rate of FRQ is temperature compensated between 22–30 °C [32]. In other cases it is apparent from the change in relative levels of clock components that reaction rates are temperature-dependent. We know that as the temperature is raised FRQ levels oscillate around a higher mean level with no change in periodicity [59]. Since levels of *frq* RNA do not change [59] this increase in FRQ protein must be due to either increased translation or increased half-life. Since FRQ positively regulates levels of WC-1, with increasing temperature one would also expect increased levels of WC-1. With more WC-1 complexing with WC-2 (which is present in excess), more FRQ might be required to repress WCC binding to the FRQ promoter. That is, because of the positive and negative actions of FRQ, increased FRQ levels might not necessarily lead to decreased period but, depending on FRQ's activity at the new temperature, the clock is self-regulatory.

**Figure 8:**
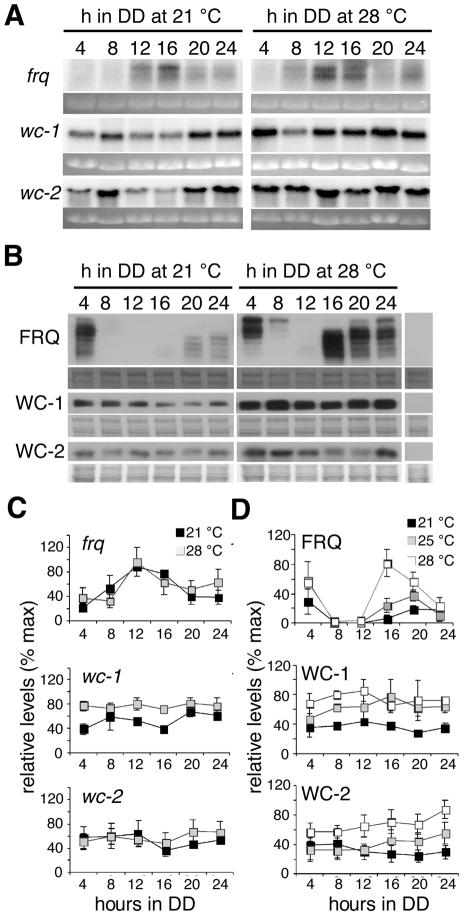
Clock components levels at 21 and 28°C. (A) Northern blot analysis of clock-specific transcript levels in strains grown in DD at either 21°C (left panel) or 28°C (right panel). Mycelial discs from wild-type or *wc1-myc* were grown in liquid culture at 21 or 28°C and tissue harvested at four-hour intervals in the first day of DD. Ethidium bromide stained gels were used to correct for loading. (B) The effects of temperature on clock-specific protein levels. Western blot analysis of clock-specific protein levels in strains grown in DD at either 21°C (left panel), 25°C (representative blot not shown) or 28°C (right panel). Amido black stained membranes were used to correct for loading. (C) Quantitative analysis of Northern blot data shown in A. Maximum transcript levels were set to 100%. (D) Quantitative analysis of western blot data shown in B. Maximum protein levels were set to 100%. The graphs in (C) and (D) represent three independent experiments. Error bars indicate ±1 standard error.

To date the effects of temperature on the *Neurospora* circadian clock have centred on the regulation of FRQ [59] and more recently on VVD [49] but there is no dataset that includes the effect of temperature on the products of the blue-light photoreceptor WC-1 and its interaction partner WC-2. To reveal the extent to which these clock components are affected by temperature, we assayed their levels over 24 hours at different temperatures. We first assayed *frq*, *wc-1* and *wc-2* transcript levels at 21 and 28°C at 4 hour intervals (Figure 8). As previously reported [59] *frq* RNA levels were not significantly different at different temperatures and peaked at DD12 (CT 5). Levels of *wc-1* RNA are lower in the first half of the day at 21°C compared to 28°C, with significant differences observed between temperatures at DD4 and DD16. *wc-2* RNA levels were comparable at most time points between 21 and 28°C. Confirming published work [41], [59] we found that peak FRQ levels were significantly higher at higher temperatures and the phosphorylation pattern of FRQ, represented by the distance the bands travel on the gel, changed throughout the day (Figure 8). At DD4, FRQ levels at all temperatures were high and the majority of FRQ is highly phosphorylated. At DD8 and DD12 FRQ levels trough at all temperatures investigated and begin to rise again at DD16. At this time hypophosphorylated forms of FRQ are detected. WC-1 levels were not found to be consistently rhythmic over time at any of the temperatures investigated. A trend towards WC-1 levels being higher at higher temperatures was observed but these differences were not significant (Figure 8D). Large and small isoforms of WC-2 [17] are detected by western blot. WC-2 levels are lower at 21°C compared to 25 and 28°C. This change in WC-2 can be incorporated into our model without affecting clock properties (data not shown).

**Figure 9:**
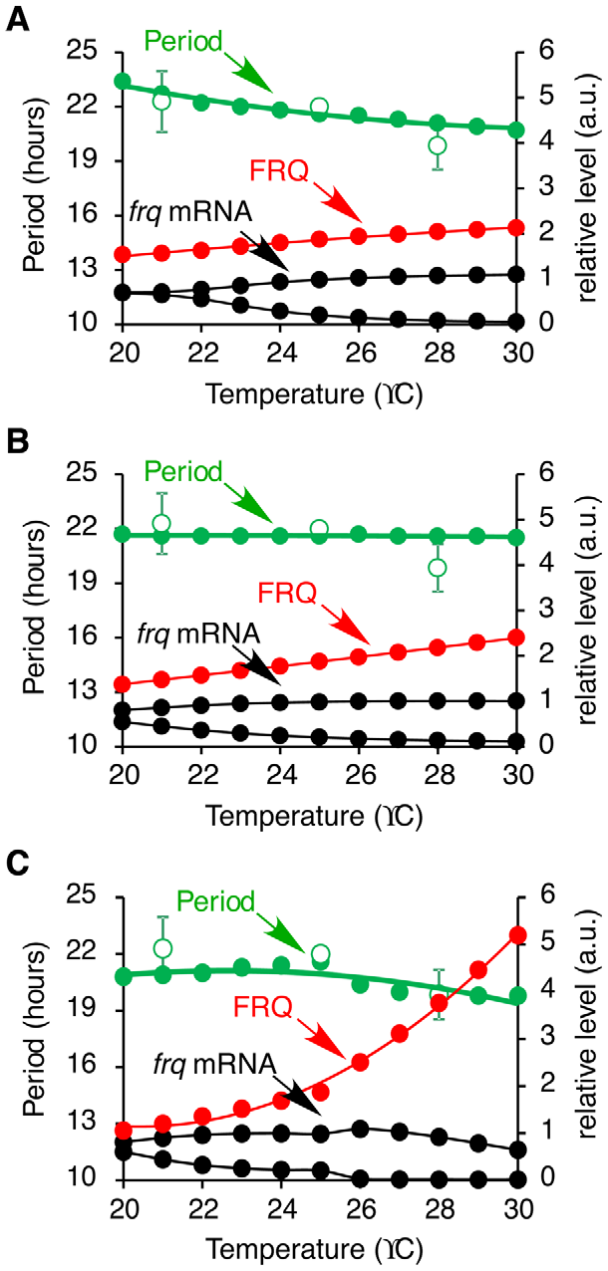
Simulated results showing the clock period and *frq* RNA and FRQ protein levels between 20 and 30°C. In (A) Only *frq* translation rate changes with temperature. In (B) translation of *frq* and the translocation of FRQ into the nucleus are temperature-dependent. (C) The translation of *frq* and the translocation of FRQ into the nucleus are temperature-dependent and *frq* translation has different activation energies above and below 25°C. Simulated period = closed green circles, experimentally derived period = open green circles. Peak levels of FRQ (closed red circles) and peak and trough levels of *frq* mRNA (closed black circles).

From experimental data, the level of *frq* mRNA remains the same at different temperatures, but the peak level of FRQ is tripled from 21 to 28°C [59] (Figure 8). However, the FRQ degradation rate remains the same over this range of temperature [32]. This finding indicates that the translation rate of *frq*, *k_FRQ*, is increased at high temperature. Figure 9A shows the results of a simulation where the rate of *frq* translation is temperature-dependent and the activation energy is 25.7 kJ/mol. Since *k_FRQ* is a negative response coefficient parameter, the increase of *frq* translation results in a shorter period (Figure 9A). FRQ can have both positive and negative regulatory effects in the model. A slight increase in *frq* translation elevates the level of *frq* mRNA and FRQ protein because FRQ positively regulates *wc-2* transcription and increases the accumulation of WCC in the nucleus. Nevertheless, a further increase of *frq* translation results in a reduction of *frq* mRNA and FRQ levels because FRQ facilitates the inactivation of WCC (data not shown). To maintain the same level of *frq* mRNA at different temperatures and accumulate FRQ at higher temperatures we investigated the effect of regulating FRQ nuclear localisation. The period response coefficient table shows that the transport rate of FRQ into the nucleus, *kin_hypoFRQc*, is a negative response coefficient parameter as is *k_FRQ*. Thus, decreasing *kin_hypoFRQc* increases the period of the clock which counterbalances the effect on period of increasing *k_FRQ*. This is consistent with the concept that temperature compensation can be achieved with balancing positive and negative contributions [54], [56]. Therefore, we hypothesise that with increasing temperature the translation of FRQ protein increases and the first order reaction rate of FRQ nuclear import decreases. Simulation results show that temperature compensation can be achieved with this hypothesis (Figure 9B). In this simulation, the activation energies of *frq* translation and FRQ nuclear localisation are 44.2 kJ/mol and −40.4 kJ/mol, respectively. The level of *frq* mRNA remains almost constant between 20–30°C and FRQ level increases as temperature increases. However, FRQ level is not tripled from 21–28°C, but as shown in Figure 8 the fold increased in FRQ level is greater between 25–28°C than between 21–25°C, indicating that the dependence of *frq* translation on temperature is not linear (Figure 8D). This observation led us to introduce different activation energies below and above 25°C for *frq* translation. A similar observation was made by Hong *et al.* [31] who noted that curved Arrhenius plots are needed in order to describe the temperature dependency of some kinetic parameters. As a result, the FRQ level is now tripled from 21–28°C and doubled from 25–28°C (Figure 9C). In this simulation, the activation energy of FRQ nuclear localisation (*kin_hypoFRQc*) is −107 kJ/mol, and the activation energies of *frq* translation (*k_FRQ*) below and above 25°C are 86.7 kJ/mol and 305.7 kJ/mol, respectively. Temperature compensation of period is achieved and the level of *frq* mRNA remains nearly constant within 20–30°C, in agreement with experimental observations.

## Discussion

Modelling of circadian clocks is an effective approach to investigate the network properties of the underlying oscillators and to understand how they interact with the environment. For example, modelling the phosphorylation of different sites of the mammalian clock protein PER showed that PER phosphorylation by casein kinase CKI can explain the period decrease and phase advance associated with some mood disorders [60]. A recent model of the *Arabidopsis* circadian clock depicts the complexity of the clock and predicted a critical role for PRR5 (Pseudo response regulator 5) in the clock-control of morning gene expression [61]. In cyanobacteria, whose circadian clock can be reconstituted *in vitro*, modelling of the oscillations of two populations of Kai proteins, including the phosphorylation/dephosphorylation of KaiC and monomer reshuffling between KaiC hexamers, has provided insight into the mechanism by which clock time is maintained when new hypophosphorylated Kai proteins are synthesized [62]. In this work, we constructed a comprehensive *Neurospora* circadian clock model and successfully simulated clock component oscillations with accurate relative levels and phases of clock components. Light responses such as phase resetting by light, entrainment to a light dark cycle and photoadaptation are also successfully predicted by the model. This model allows us to postulate that temperature compensation is achieved by a concomitant increase in *frq* translation and inhibition of FRQ nuclear localisation.

In constant darkness, transcriptional regulation of *frq* by the WCC is a key step in *Neurospora*'s circadian oscillator and periodic transcription by WCC is dependent on its phosphorylation state [16]. Whereas hypophosphorylated WCC is degraded after activating *frq* transcription, phosphorylated WCC is more stable and can be shuttled out of the nucleus into the cytoplasm where dephosphorylation may occur [14]. The latter pathway results in the reappearance of hypophosphorylated WCC in the nucleus, which ensures the availability of WCC for the next cycle of transcription activation. Our model recapitulates these experimental observations: degradation of WCC as a consequence of its role as a transcriptional activator of *frq* results in the reduction of WCC during the increasing phase of *frq* transcription. Furthermore, when FRQ levels peak, phosphorylation of the WCC, promoted by rising levels of FRQ, allows the complex to escape degradation and enter the cycle of nuclear cytoplasmic translocation. As levels of WCC reach their zenith, FRQ gradually decreases because of the decreased activation of *frq* transcription by WCC. Thus, WCC promotes *frq* transcription when the level of FRQ is low. Another possible pathway leading to the degradation of WCC centres on WCC phosphorylation state. We tested this hypothesis by modelling WCC degradation via phosphorylation. In this case our model predicts that when FRQ decreases, a delay occurs before the synthesis of new WCC and no antiphasic behaviour between WCC and FRQ is seen. Consequently, our model supports the hypothesis that degradation of WCC via transcription activation is a key factor for antiphasic FRQ and WCC expression. A putative WCC binding site exists in the *wc-1* promoter [35] but a mutation of WC-2 Zn finger DNA-binding domain does not affect the expression of WC-1 [14] indicating that a transcription factor other than the WCC regulates expression of *wc-1*. The model shows that the inferred transcription factor is necessary for antiphasic expression of WC-1 and FRQ.

An important aspect of circadian clocks is their ability to integrate signals from their environment. When exposed to light *frq* mRNA level is elevated and variable [45], FRQ is maintained at approximately twice the level seen in the dark [48] and the *Neurospora* oscillator dampens [48]. On transfer from light to dark, *frq* RNA and protein are degraded and rhythmic expression of *frq* resumes [41], [47]. Our model incorporates a mechanism by which VVD-mediated inactivation of WCC reduces *frq* mRNA transcription at the light to dark boundary as well as the role of the FRQ-FRH complex in facilitating *frq* mRNA degradation in a FRQ concentration dependent manner [23], [24], [25], [47], [63]. In our model both mechanisms are essential and sufficient for initiating the oscillation after the light to dark transfer.

The least understood characteristic of circadian clocks, namely temperature compensation of circadian rhythmicity, has been a focus of this study. Temperature compensation may be achieved in a number of different ways, involving either true or apparent constancy of reaction rates. For example, a seemingly temperature insensitive reaction rate has been reported for the phosphorylation of the mammalian clock protein PER2 by CK1ε and CK1δ [64] and this is thought to be important for temperature compensation. On the other hand, in *Synechococcus* the phosphorylation cycle of KaiC depends on, but also influences, the protein's ATPase activity. Whilst the ATPase activity of KaiC is apparently temperature compensated, this is not an inherent property of the ATPase but is thought to be due to an inbuilt feedback inhibition in KaiC that downregulates its ATPase activity with increasing temperature due to a conformational change in the KaiC hexamer itself. It is speculated that the energy produced from the hydrolysis of ATP is held by KaiC resulting in an altered conformation of the protein that counterbalances changes in its activity [65]. More complex regulations in clock protein activities have been proposed in *Arabidopsis*, where not just one but many different clock components apparently participate to various degrees in the oscillation at different temperatures [66], [67].

In *Neurospora* and in other organisms mutant phenotypes indicate a role for both transcriptional and posttranscriptional processes in temperature compensation [10]. Genetic evidence indicates that mutations in the known *Neurospora* clock components *frq*, *wc-2*, *chrono (chr)* and *period-3 (prd-3)* alter temperature compensation properties over a range of temperatures [44], [68]. Theoretically, transcriptional and posttranscriptional processes that act to increase or decrease period in a temperature-dependent way could feed into temperature compensation. For instance, the phosphorylation state of FRQ and the WHITE COLLAR proteins dictates their activity and stability and mutations or chemicals affecting FRQ stability affect temperature compensation [69], [70], [71], [72], [73]. Because FRQ stability is regulated by kinases and phosphatases one would predict that the action of these enzymes will be integral to successful temperature compensation of the clock [74] and this is indeed true [32], [75]. The apparent temperature compensation of FRQ degradation rate is brought about by different utilization of FRQ phosphorylation sites at different temperatures. Differential accessibility of FRQ phosphorylation sites is probably regulated by a conformational change in FRQ with changing temperature [32]. In addition, our data imply that temperature-dependent changes in the localization of FRQ are an important aspect of temperature compensation.

To be valid, the proposed mechanism of temperature compensation has to be in agreement with a certain number of experimental observations. Between 21 and 28°C: (1) *frq* RNA levels are unchanged, (2) FRQ protein levels increase by 3–4 fold, (3) the degradation rate of FRQ is unchanged. Thus, the increase in FRQ levels must be due to an increase in *frq* translation. However, if the *frq* translation rate is increased significantly, the model predicts that the *frq* RNA level would decrease and the period shorten. Hence, the increase in *frq* translation has to be compensated by other reactions that: (1) increase the period, (2) increase the *frq* RNA level, and (3) triple the FRQ level. We first tested individual reaction parameters to see whether these conditions could be fulfilled over a range of temperatures. When we considered a fixed activation energy for *frq* translation, no parameter variations could fulfil all three conditions and the oscillation was lost at lower temperatures. We then considered different activation energies below and above 25°C for *frq* translation. The reason why *frq* translation might have different activation energies may lie in the complexity of the mechanisms of translation where multiple enzymes are involved. In addition, the structure of mRNA and the conformation of FRQ may be changed at different temperatures. Similarly, Hong *et al.* [31] employed a curved Arrhenius plot for WCC binding to the *frq* promoter and the degradation of FRQ, and used different activation energies depending on the range of temperature (20–25 °C or 25–30 °C) to explain temperature compensation. Using different activation energies for *frq* translation, we observed that when: (1) the Michaelis constant of nuclear WCC phosphorylation is increased with increasing temperature, or (2) the nuclear localisation of FRQ is decreased with increasing temperature, the three conditions are fulfilled. However, the second modification leads to simulations that are in better agreement with experimental data. Temperature dependent translocation of proteins has been reported in other organisms, for example in rat fibroblasts the temperature-sensitive mutant of p53 (p53^val-5^) is predominantly in the cytoplasm at 37.5 °C but moves to the nucleus at 32.5 °C [76]. Another example of a protein whose localisation is temperature-dependent is the *Antirrhinum* Tam3 transposase, which is restricted to the cytoplasm at 25 °C, but translocates into the nucleus at 15 °C [77]. Interestingly, translocation between cytoplasm and nucleus of the *Drosophila* clock protein PER is restricted in the *ritsu* mutant at higher temperatures, resulting in lengthening the period of the clock [78].

How might subcellular localization of FRQ be regulated? Nuclear translocation may be regulated by FRQ phosphorylation, although more recent evidence suggests that this is not the case and that at least at 25 °C, FRQ's interaction with FRH and overall conformation plays a greater role [79]. This being true one would predict that some other posttranslational modification and/or change in conformation of FRQ regulates nuclear localization with temperature. Our proposed temperature compensation mechanism could be tested by carrying out FRQ subcellular distribution experiments. If our prediction is correct, nuclear localisation of FRQ should be restricted at high temperature. If the ratio of cytoplasmic FRQ to nuclear FRQ is similar at low and high temperature, this would suggest that although FRQ level is essentially increased, the activity of FRQ may be diminished at higher temperature, ensuring that levels of WCC phosphorylation are maintained.

In addition to temperature-regulated FRQ nuclear localization, other posttranscriptional modifications may be required to maintain constant levels and activities of the clock components which seem to be unaffected by the temperature. For example, overall WCC activity appears to stay the same at all temperatures, resulting in similar levels of cycling *frq* RNA. However, this may be due to modification of WC-1 at higher temperatures which renders it less active. Indeed, the WC-1 protein runs at a higher molecular weight at 28°C. What could be the significance of the unchanged levels of *frq* mRNA? We speculate that the purpose of keeping *frq* RNA levels constant could be that transcription is the entry point for resetting of the *Neurospora* clock by light, and possibly also temperature. It seems plausible that in order to retain the ability to respond to light at different temperatures, the transcriptional responsiveness has to be maintained at subsaturated levels at all times. FRQ levels increase with temperature yet we predict much of the FRQ protein is excluded from the nucleus. One reason for this could be that a non circadian function of FRQ in the cytoplasm requires increased FRQ at higher temperatures. Additionally, when temperatures drop high levels of FRQ help to reset the clock to the appropriate circadian time [47], [59].

In summary, we provide a comprehensive model for the *Neurospora* circadian clock and its responses to acute and chronic changes in light and temperature. The model predicts a role for FRQ nuclear localisation in temperature compensation and makes predictions that can be experimentally tested in the future to further refine our understanding of circadian oscillators. Both light and temperature can entrain and reset the clock [50], [52], [59], and in reality organisms are exposed to these conditions simultaneously. An advantage of having a model that incorporates both light response and temperature dependency is that the coupling between both types of environmental signals can be studied to provide a comprehensive understanding of the detailed molecular interactions of the clock.

## Materials and Methods

### Model construction

The circadian clock model was manually constructed using the CellDesigner™ 4.1 software [80], [81], [82]. The program was operated in Java Runtime Environment (JRE) Version 6 Update 16 on Windows XP. Model parameters were either derived from experimental data (*frq* ([63] and our data), *wc-1* (our data), *wc-2* (our data) and *vvd* RNA degradation (our data), FRQ degradation [30]) or estimated by comparing simulations to experimental observations. The kinetic equations used in the model are presented in Table S1 and the model is provided in SBML format Level 2 version 4 (Dataset S1). Simulations were carried out in CellDesigner using SOSlib as the numerical solver.

### Control analysis

To quantify how model components affect the period and amplitude of the oscillation, we calculated period and amplitude response coefficients. The period response coefficient *R_j_^T^* of parameter *P_j_* was defined as the rate of change in period *T* divided by the rate of change of the parameter value.
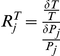
The amplitude response coefficient *R_j_^A^* of the parameter *P_j_* was similarly defined as the rate of change in amplitude *A* divided by the rate of change of the parameter value.
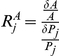
The effect of a 3% change in the value of each parameter was considered. 200 hours of dark simulation and 200 points per hour were calculated without changing the initial value of the components for period response coefficients. 200 hours of dark simulation and 50 points per hour are calculated without changing the initial value of the components for amplitude response coefficients. The last two peaks of *frq* mRNA level were used to calculate the period and the last peak and trough of the *frq* mRNA oscillation were used to calculate the amplitude.

### Light input and photoadaptation

Light activated WCC (laWCC) was introduced in the model and was activated from nuclear hypophosphorylated WCC (hypoWCCn). laWCC can activate the transcription of *vvd* and promote the transcription of *frq* and *wc-1*. The light activation rate (*klact_hypoWCCn*) of WCC is 0 in dark and is increased to 5 to mimic light using SBML events. Photoadaptation occurs because light induced VVD represses the light activity of the WCC through the formation of the laWCC VVD complex (WVC). After interacting with VVD the laWCC disassociates and returns to its dark state.

For phase response curves (PRC) simulated in the model, 0.1 or 0.01 h of duration of light was pulsed every two circadian hours. To simulate a light pulse, the light activation rate (*klact_hypoWCCn*) of WCC was changed from 0 to 5, and returned to 0 to end the pulse. The time of peak *frq* mRNA before and after the light pulse was used to calculate the advance or delay of the clock.

### Temperature compensation

The effect of temperature was introduced into the model by use of the Arrhenius equation [27]. For each reaction, temperature influences the value of each kinetic parameter *k_i_* according to equation 1 [30].
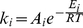
(1)
*A_i_* is the collision factor or pre-exponential factor, which is a constant; *E_i_* is the activation energy; *R* is the gas constant (8.314462 J·mol^−1^·K^−1^); *T* is the temperature in Kelvin. *A_i_* and *E_i_* are independent of the temperature. The activation energy *E_a_* was calculated using equation 2 [30].
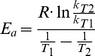
(2)
*R* is the gas constant *k_T1_* is the parameter value at temperature *T_1_* and *k_T2_* is the parameter value at temperature *T_2_*. Once the activation energy was calculated, the pre-exponential factor *A_i_* was obtained by solving the Arrhenius equation.

While all reactions are temperature-dependent in principle, the reactions that are explicitly made temperature-dependent in the model are sufficient to account for all observations related to temperature change, and as such they represent the most likely mechanism for temperature compensation. All other reactions could indeed be made explicitly temperature-dependent in the model, but their activation energies would remain too small to have any noticeable effect on simulations. This would mean that unnecessary complexity is added to the model that cannot be validated by observations, thereby contravening the principle of parsimony. The activation energy of FRQ nuclear localisation (*kin_hypoFRQc*) is −107 kJ/mol, and the activation energies of *frq* translation (*k_FRQ*) below and above 25°C are 86.7 kJ/mol and 305.7 kJ/mol, respectively.

### Model robustness to parameter perturbation

The model robustness analysis was carried out using the parameter scan function in CellDesigner. For the rhythmicity test, each parameter was increased and decreased until the oscillation of *frq* RNA level was lost. 20,000 hours of dark simulation and 10 points per hour were calculated without changing the initial value of the components. A persistent oscillation of *frq* mRNA level was considered as rhythmic. For the period perturbation test, each parameter was increased and decreased until the period lay outside the range of 21.6±0.6 hours. For the amplitude perturbation test, each parameter was increased and decreased until the *frq* RNA oscillation amplitude was increased or decreased by 5% of its original value. For the period and the amplitude perturbation test, 500 hours of dark simulation and 10 points per hour were calculated without changing the initial value of the components. The last two peaks of *frq* mRNA level were used to calculate the period and the last peak and trough of the *frq* mRNA oscillation were used to calculate the amplitude.

### Strains and conditions

Minimal medium contained 1× Vogel's salts [83], 2% sucrose, 1.5% agar and 50 ng/ml biotin. Liquid medium consisted of 1× Vogel's salts, 2% glucose, 50 ng/ml biotin and 0.17% arginine. mRNA degradation was assayed in the 54-3 *bd* strain of *Neurospora*. *Neurospora* was grown on slant minimal medium and spores were transferred to plate with liquid medium. After 24 hours culture at 30 °C, tissues were cut into discs and inoculated into flasks with liquid medium. Flasks were shaken at 125 rpm. Discs were grown in shake culture in constant light (LL) for at least 24 hours. At the time point of light to dark (DD) transfer, thiolutin was dissolved in dimethyl sulfoxide (DMSO) and added to a final concentration of 5 µg/ml. To assay mRNA and protein expression in constant conditions but at different ambient temperatures, the 54-3 *bd* or the *wc1-myc* strain of *Neurospora* were grown for 1–2 days at either 21°C, 25°C or 28°C in LL and then transferred to DD at the same constant temperature.

### Northern blot analysis

Transcripts were extracted by using the Qiagen RNeasy Mini kit according to the manufacturer's instructions for the isolation of total RNA from filamentous fungus. Total RNA (7–10 µg) was electrophoresed through a 1% agarose–formaldehyde gel, blotted onto Hybond-N+ membrane (Amersham), and probed using radiolabelled antisense riboprobes (Ambion) as described previously [48], [49]. Nucleotides 1630–3832 of the *frequency* open reading frame (ORF) were transcribed into an antisense riboprobe using Amersham ^32^P-dUTP (800 Ci/mmol) to a specific activity of 10^9^ counts per minute (cpm) per microgram. For *wc-1* (positions 1756–3067) and *wc-2* (positions 637–1801) gene specific riboprobes were generated by labelling PCR fragments containing T7 polymerase sites to generate antisense riboprobes. Gene-specific riboprobes of *vvd* mRNA were obtained by labelling PCR products (AF338412, positions 239–1173 for *vvd*) containing an appropriate T7 Polymerase site to generate antisense riboprobes. Membranes were hybridized in 10 ml of NorthernMax Prehyb/Hyb (Ambion) containing 2×10^7^ cpm/ml of *in vitro* transcribed radiolabelled probe (Ambion). Membranes were exposed to Fuji screens and were scanned using a PhosphorImager (Bio-Rad). RNA data were quantified using ImageJ 1.42q (National Institutes of Health, USA) or Quantity One (Bio-Rad).

### Protein analysis

Total protein extracts were obtained as previously described [41]. For western blot analysis, 50 µg of total protein extract was loaded per lane onto an SDS-PAGE gel. After electrophoresis, proteins were blotted onto Immobilon-P membrane (Millipore) by wet transfer. Membranes were hybridised with either anti-FRQ (kindly provided by Prof. Jay Dunlap and Prof. Jennifer Loros, Dartmouth Medical School, Hanover, NH), anti-WC-2 (kindly provided by Prof. Yi Liu, University of Texas Southwestern Medical School, Dallas, TX) or anti-MYC antibody (Santa Cruz Biotechnology) as described previously [22]. Immunodetection was carried out as previously described [49] and the signal quantified using Quantity One (Bio-Rad).

### Race tube assay

The period of the clock was assayed in the 54-3 *bd* strain of *Neurospora* at 21°C, 25°C and 28°C. Race tube media contained 1× Vogel's salts, 0.1% glucose, 50 ng/µL biotin, 0.17% arginine, and 1.5% agar. *Neurospora* were grown in constant light (LL) for at least 24 hours and then transferred to DD at the same constant temperature. Growth fronts were first marked at the LD transition and then every 24 h thereafter. All race tubes were analyzed using the CHRONO program [84].

## Supporting Information

Dataset S1 ***Neurospora*** **circadian clock model in SBML format Level 2 version 4 for CellDesigner.**
(XML)

Figure S1 **Period response coefficients.** Values of averaged period response coefficients for ±3% variation in each parameter value. 200 points per hour and 200 hours in total were simulated. The last two peaks of *frq* mRNA were used to determine the period.
(DOC)

Figure S2 **Amplitude response coefficients.** Values of averaged amplitude response coefficients for ±3% variation in each parameter value. 50 points per hour and 200 hours in total were simulated. The last peak and trough of *frq* mRNA were used to determine the amplitude.
(DOC)

Table S1 **Kinetic equations used in the model.** The rate of reaction *i* is noted *v_i*.
(DOC)

Table S2 **List and values of model parameters.**
(DOC)

Table S3 **Parameter sensitivity test for oscillations.** For each parameter, the table gives the lower and upper value that conserves *frq* RNA oscillations, as well as the percentage change with respect to its reference value.
(DOC)

Table S4 **Parameter sensitivity test for period.** For each parameter, the table gives the lower and upper value for which a period of 21.6±0.6 hours is obtained for *frq* RNA oscillations, as well as the percentage change with respect to its reference value.
(DOC)

Table S5 **Parameter sensitivity test for amplitude.** For each parameter, the table gives the lower and upper value for which the amplitude of *frq* RNA of oscillations is changed by ±5%, as well as the percentage change with respect to its reference value.
(DOC)

## References

[1] Bell-Pedersen D, Cassone VM, Earnest DJ, Golden SS, Hardin PE (2005). Circadian rhythms from multiple oscillators: lessons from diverse organisms.. Nat Rev Genet.

[2] Hunt T, Sassone-Corsi P (2007). Riding tandem: circadian clocks and the cell cycle.. Cell.

[3] Harrisingh MC, Nitabach MN (2008). Circadian rhythms. Integrating circadian timekeeping with cellular physiology.. Science.

[4] Ko GY, Shi L, Ko ML (2009). Circadian regulation of ion channels and their functions.. J Neurochem.

[5] Heintzen C, Liu Y (2007). The *Neurospora crassa* circadian clock.. Adv Genet.

[6] Dunlap JC, Loros JJ, Colot HV, Mehra A, Belden WJ (2007). A circadian clock in *Neurospora*: how genes and proteins cooperate to produce a sustained, entrainable, and compensated biological oscillator with a period of about a day.. Cold Spring Harb Symp Quant Biol.

[7] Pittendrigh CS (1954). On Temperature Independence in the Clock System Controlling Emergence Time in *Drosophila*.. Proc Natl Acad Sci U S A.

[8] Dunlap JC (1999). Molecular bases for crcadian clocks.. Cell.

[9] Dong G, Golden SS (2008). How a cyanobacterium tells time.. Curr Opin Microbiol.

[10] Zheng X, Sehgal A (2008). Probing the relative importance of molecular oscillations in the circadian clock.. Genetics.

[11] Dardente H, Cermakian N (2007). Molecular circadian rhythms in central and peripheral clocks in mammals.. Chronobiol Int.

[12] Loros JJ, Dunlap JC (2001). Genetic and molecular analysis of circadian rhythms in *Neurospora*.. Annu Rev Physiol.

[13] Cheng P, Yang Y, Liu Y (2001). Interlocked feedback loops contribute to the robustness of the *Neurospora* circadian clock.. Proc Natl Acad Sci U S A.

[14] Schafmeier T, Diernfellner A, Schafer A, Dintsis O, Neiss A (2008). Circadian activity and abundance rhythms of the *Neurospora* clock transcription factor WCC associated with rapid nucleo-cytoplasmic shuttling.. Genes Dev.

[15] He Q, Cha J, Lee HC, Yang Y, Liu Y (2006). CKI and CKII mediate the FREQUENCY-dependent phosphorylation of the WHITE COLLAR complex to close the *Neurospora* circadian negative feedback loop.. Genes Dev.

[16] Schafmeier T, Haase A, Káldi K, Scholz J, Fuchs M (2005). Transcriptional feedback of *Neurospora* circadian clock gene by phosphorylation-dependent inactivation of its transcription factor.. Cell.

[17] Neiss A, Schafmeier T, Brunner M (2008). Transcriptional regulation and function of the *Neurospora* clock gene *white collar 2* and its isoforms.. EMBO Rep.

[18] Chen CH, Dunlap JC, Loros JJ (2010). *Neurospora* illuminates fungal photoreception.. Fungal Genet Biol.

[19] Linden H, Macino G (1997). White collar 2, a partner in blue-light signal transduction, controlling expression of light-regulated genes in *Neurospora crassa*.. EMBO J.

[20] Froehlich AC, Liu Y, Loros JJ, Dunlap JC (2002). White Collar-1, a circadian blue light photoreceptor, binding to the *frequency* promoter.. Science.

[21] Schwerdtfeger C, Linden H (2003). VIVID is a flavoprotein and serves as a fungal blue light photoreceptor for photoadaptation.. EMBO J.

[22] Heintzen C, Loros JJ, Dunlap JC (2001). The PAS protein VIVID defines a clock-associated feedback loop that represses light input, modulates gating, and regulates clock resetting.. Cell.

[23] Hunt SM, Thompson S, Elvin M, Heintzen C (2010). VIVID interacts with the WHITE COLLAR complex and FREQUENCY-interacting RNA helicase to alter light and clock responses in *Neurospora*.. Proc Natl Acad Sci U S A.

[24] Chen CH, DeMay BS, Gladfelter AS, Dunlap JC, Loros JJ (2010). Physical interaction between VIVID and white collar complex regulates photoadaptation in *Neurospora*.. Proc Natl Acad Sci U S A.

[25] Malzahn E, Ciprianidis S, ldi KK, Schafmeier T, Brunner M (2010). Photoadaptation in *Neurospora* by competitive interaction of activating and inhibitory LOV domains.. Cell.

[26] Ruoff P, Mohsenzadeh S, Rensing L (1996). Circadian rhythms and protein turnover: the effect of temperature on the period lengths of clock mutants simulated by the Goodwin oscillator.. Naturwissenschaften.

[27] Ruoff P, Rensing L (1996). The temperature compensated Goodwin model simulates many circadian clock properties.. J Theor Biol.

[28] Leloup J-C, Gonze D, Goldbeter1 A (1999). Limit cycle models for circadian rhythms based on transcriptional regulation in *Drosophila* and *Neurospora*.. J Biol Rhythms.

[29] Francois P (2005). A model for the *Neurospora* circadian clock.. Biophys J.

[30] Ruoff P, Loros JJ, Dunlap JC (2005). The relationship between FRQ-protein stability and temperature compensation in the *Neurospora* circadian clock.. Proc Natl Acad Sci U S A.

[31] Hong CI, Jolma IW, Loros JJ, Dunlap JC, Ruoff P (2008). Simulating dark expressions and interactions of *frq* and *wc-1* in the *Neurospora* circadian clock.. Biophys J.

[32] Mehra A, Shi M, Baker CL, Colot HV, Loros JJ (2009). A role for Casein Kinase 2 in the mechanism underlying circadian temperature compensation.. Cell.

[33] Dunlap JC, Loros JJ (2006). How fungi keep time: circadian system in *Neurospora* and other fungi.. Curr Opin Microbiol.

[34] Froehlich AC, Loros JJ, Dunlap JC (2003). Rhythmic binding of a WHITE COLLAR-containing complex to the *frequency* promoter is inhibited by FREQUENCY.. Proc Natl Acad Sci U S A.

[35] Káldi K, González BH, Brunner M (2006). Transcriptional regulation of the *Neurospora* circadian clock gene *wc-1* affects the phase of circadian output.. EMBO Rep.

[36] Cheng P, He Q, He Q, Wang L, Liu1 Y (2005). Regulation of the *Neurospora* circadian clock by an RNA helicase.. Genes Dev.

[37] Diernfellner ACR, Querfurth C, Salazar C, Höfer T, Brunner M (2009). Phosphorylation modulates rapid nucleocytoplasmic shuttling and cytoplasmic accumulation of *Neurospora* clock protein FRQ on a circadian time scale.. Genes Dev.

[38] Brunner M, Schafmeier T (2006). Transcriptional and post-transcriptional regulation of the circadian clock of cyanobacteria and *Neurospora*.. Genes Dev.

[39] Ballario P, Vittorioso P, Magrelli A, Talora C, Cabibbo A (1996). White collar-1, a central regulator of blue light responses in *Neurospora*, is a zinc finger protein.. EMBO J.

[40] He Q, Cheng P, Yang Y, Wang L, Gardner KH (2002). White collar-1, a DNA binding transcription factor and a light sensor.. Science.

[41] Garceau NY, Liu Y, Loros JJ, Dunlap JC (1997). Alternative initiation of translation and time-specific phosphorylation yield multiple forms of the essential clock protein FREQUENCY.. Cell.

[42] Lee K, Loros JJ, Dunlap JC (2000). Interconnected feedback loops in the *Neurospora* circadian system.. Science.

[43] Denault DL, Loros JJ, Dunlap JC (2001). WC-2 mediates WC-1-FRQ interaction within the PAS protein-linked circadian feedback loop of *Neurospora*.. EMBO J.

[44] Collett MA, Dunlap JC, Loros JJ (2001). Circadian clock-specific roles for the light response protein WHITE COLLAR-2.. Mol Cell Biol.

[45] Crosthwaite SK, Loros JJ, Dunlap JC (1995). Light-induced resetting of a circadian clock is mediated by a rapid increase in *frequency* transcript.. Cell.

[46] Merrow M, Franchi L, Dragovic Z, Gorl M, Johnson J (2001). Circadian regulation of the light input pathway in *Neurospora crassa*.. EMBO J.

[47] Guo J, Cheng P, Yuan H, Liu Y (2009). The exosome regulates circadian gene expression in a posttranscriptional negative feedback loop.. Cell.

[48] Elvin M, Loros JJ, Dunlap JC, Heintzen C (2005). The PAS/LOV protein VIVID supports a rapidly dampened daytime oscillator that facilitates entrainment of the *Neurospora* circadian clock.. Genes Dev.

[49] Hunt SM, Elvin M, Crosthwaite SK, Heintzen C (2007). The PAS/LOV protein VIVID controls temperature compensation of circadian clock phase and development in *Neurospora crassa*.. Genes Dev.

[50] Johnson CH (1999). Forty years of PRCs–what have we learned?. Chronobiol Int.

[51] Sargent ML, Briggs WR (1967). The effects of light on a circadian rhythm of conidiation in *Neurospora*.. Plant Physiol.

[52] Dharmananda S (1980). Studies of the circadian clock of *Neurospora crassa*: light-induced phase shifting..

[53] Hastings JW, Sweeney BM (1957). On the Mechanism of Temperature Independence in a Biological Clock.. Proc Natl Acad Sci U S A.

[54] Winfree AT (1980). The geometry of biological time.

[55] Ruoff P (1992). Introducing temperature-compensation in any reaction kinetic oscillator model.. J Interdiscipl Cycle Res.

[56] Ruoff P, Zakhartsev M, Westerhoff HV (2007). Temperature compensation through systems biology.. FEBS J.

[57] Kageyama H, Nishiwaki T, Nakajima M, Iwasaki H, Oyama T (2006). Cyanobacterial circadian pacemaker: Kai protein complex dynamics in the KaiC phosphorylation cycle in vitro.. Mol Cell.

[58] Terauchi K, Kitayama Y, Nishiwaki T, Miwa K, Murayama Y (2007). ATPase activity of KaiC determines the basic timing for circadian clock of cyanobacteria.. Proc Natl Acad Sci U S A.

[59] Liu Y, Merrow M, Loros JJ, Dunlap JC (1998). How temperature changes reset a circadian oscillator.. Science.

[60] Leloup J-C, Goldbeter A (2011). Modelling the dual role of Per phosphorylation and its effect on the period and phase of the mammalian circadian clock.. IET Syst Biol.

[61] Pokhilko A, Hodge SK, Stratford K, Knox K, Edwards KD (2010). Data assimilation constrains new connections and components in a complex, eukaryotic circadian clock model.. Mol Syst Biol.

[62] Nagai T, Terada TP, Sasai M (2010). Synchronization of circadian oscillation of phosphorylation level of KaiC in vitro.. Biophys J.

[63] Guo J, Cheng P, Liu Y (2010). Functional significance of FRH in regulating the phosphorylation and stability of *Neurospora* circadian clock protein FRQ.. J Biol Chem.

[64] Isojima Y, Nakajima M, Ukai H, Fujishima H, Yamada RG (2009). CKIepsilon/delta-dependent phosphorylation is a temperature-insensitive, period-determining process in the mammalian circadian clock.. Proc Natl Acad Sci U S A.

[65] Kondo T (2007). A cyanobacterial circadian clock based on the Kai oscillator.. Cold Spring Harb Symp Quant Biol.

[66] Gould PD, Diaz P, Hogben C, Kusakina J, Salem R (2009). Delayed fluorescence as a universal tool for the measurement of circadian rhythms in higher plants.. Plant J.

[67] Salome PA, Weigel D, McClung CR (2010). The role of the *Arabidopsis* morning loop components CCA1, LHY, PRR7, and PRR9 in temperature compensation.. Plant Cell.

[68] Gardner GF, Feldman JF (1981). Temperature compensation of circadian periodicity in clock mutants of *Neurospora crassa*.. Plant Physiol.

[69] Gorl M, Merrow M, Huttner B, Johnson J, Roenneberg T (2001). A PEST-like element in FREQUENCY determines the length of the circadian period in *Neurospora crassa*.. EMBO J.

[70] He Q, Liu Y (2005). Degradation of the *Neurospora* circadian clock protein FREQUENCY through the ubiquitin-proteasome pathway.. Biochem Soc Trans.

[71] Jolma IW, Falkeid G, Bamerni M, Ruoff P (2006). Lithium leads to an increased FRQ protein stability and to a partial loss of temperature compensation in the *Neurospora* circadian clock.. J Biol Rhythms.

[72] Liu Y, Loros J, Dunlap JC (2000). Phosphorylation of the *Neurospora* clock protein FREQUENCY determines its degradation rate and strongly influences the period length of the circadian clock.. Proc Natl Acad Sci U S A.

[73] Sancar G, Sancar C, Brunner M, Schafmeier T (2009). Activity of the circadian transcription factor White Collar Complex is modulated by phosphorylation of SP-motifs.. FEBS Lett.

[74] Sweeney BM, Hastings JW (1960). Effects of temperature upon diurnal rhythms.. Cold Spring Harb Symp Quant Biol.

[75] Tang CT, Li S, Long C, Cha J, Huang G (2009). Setting the pace of the *Neurospora* circadian clock by multiple independent FRQ phosphorylation events.. Proc Natl Acad Sci U S A.

[76] Ginsberg D, Michael-Michalovitz D, Ginsberg D, Oren M (1991). Induction of growth arrest by a temperature-sensitive p53 mutant is correlated with increased nuclear localization and decreased stability of the protein.. Mol Cell Biol.

[77] Fujino K, Hashida S-n, Ogawa T, Natsume T, Uchiyama T (2011). Temperature controls nuclear import of Tam3 transposase in *Antirrhinum*.. Plant J.

[78] Matsumoto A, Tomioka K, Chiba Y, Tanimura T (1999). timrit Lengthens circadian period in a temperature-dependent manner through suppression of PERIOD protein cycling and nuclear localization.. Mol Cell Biol.

[79] Cha J, Yuan H, Liu Y (2011). Regulation of the activity and cellular localization of the circadian clock protein FRQ.. J Biol Chem.

[80] Kitano H, Funahashi A, Matsuoka Y, Oda K (2005). Using process diagrams for the graphical representation of biological networks.. Nat Biotechnol.

[81] Funahashi A, Matsuoka Y, Jouraku A, Morohashi M, Kikuchi N (2008). CellDesigner 3.5: A versatile modeling tool for biochemical networks.. Proc IEEE Inst Electr Electron Eng.

[82] Funahashi A, Morohashi M, Kitano H, Tanimura N (2003). CellDesigner: a process diagram editor for gene-regulatory and biochemical networks.. Drug Discov Today Biosilico.

[83] Davis RH, de Serres FJ (1970). Genetic and microbial research techniques for Neurospora crassa.. Methods Enzymol.

[84] Roenneberg T, Taylor W (2000). Automated recordings of bioluminescence with special reference to the analysis of circadian rhythms.. Methods Enzymol.

