# Supplementary material for: Comprehensive Modelling of the Neurospora Circadian Clock and Its Temperature Compensation
Source: PLoS Comput Biol. 2012 Mar 29;8(3):e1002437. doi: 10.1371/journal.pcbi.1002437 (PMC3320131; doi:10.1371/journal.pcbi.1002437)
Supplement: Figure S1 — Period response coefficients. Values of averaged period response coefficients for ±3% variation in each parameter value. 200 points per hour and 200 hours in total were simulated. The last two peaks of frq mRNA were used to determine the period. (DOC) [file pcbi.1002437.s002.doc]

**Figure S1: Period response coefficients**

Values of averaged period response coefficients for ± 3 % variation in each parameter value. 200 points per hour and 200 hours in total were simulated. The last two peaks of *frq* mRNA were used to determine the period.

**
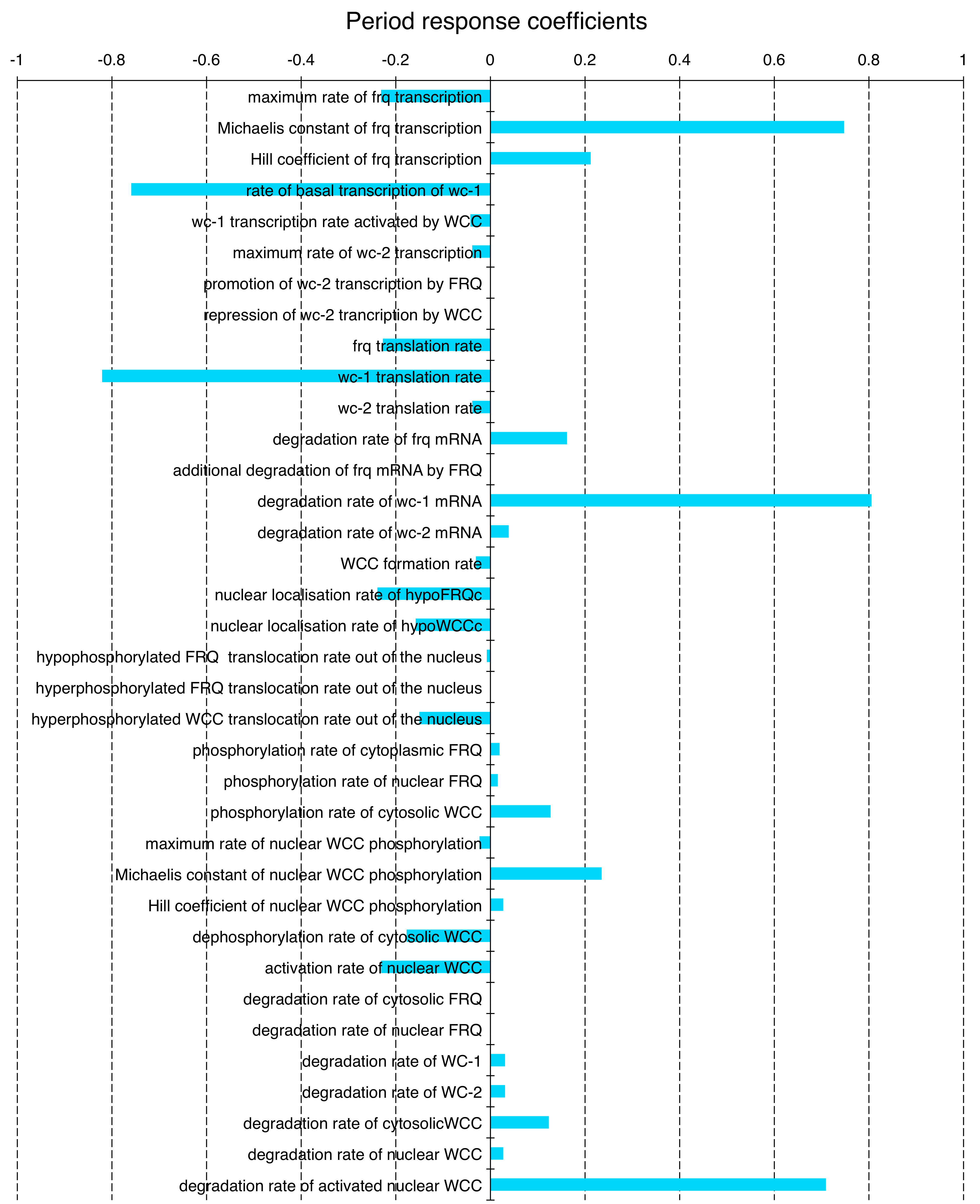
**
