# Supplementary material for: Comprehensive Modelling of the Neurospora Circadian Clock and Its Temperature Compensation
Source: PLoS Comput Biol. 2012 Mar 29;8(3):e1002437. doi: 10.1371/journal.pcbi.1002437 (PMC3320131; doi:10.1371/journal.pcbi.1002437)
Supplement: Figure S2 — Amplitude response coefficients. Values of averaged amplitude response coefficients for ±3% variation in each parameter value. 50 points per hour and 200 hours in total were simulated. The last peak and trough of frq mRNA were used to determine the amplitude. (DOC) [file pcbi.1002437.s003.doc]

**Figure S2: Amplitude response coefficients**

Values of averaged amplitude response coefficients for ± 3 % variation in each parameter value. 50 points per hour and 200 hours in total were simulated. The last peak and trough of *frq* mRNA were used to determine the amplitude.

**
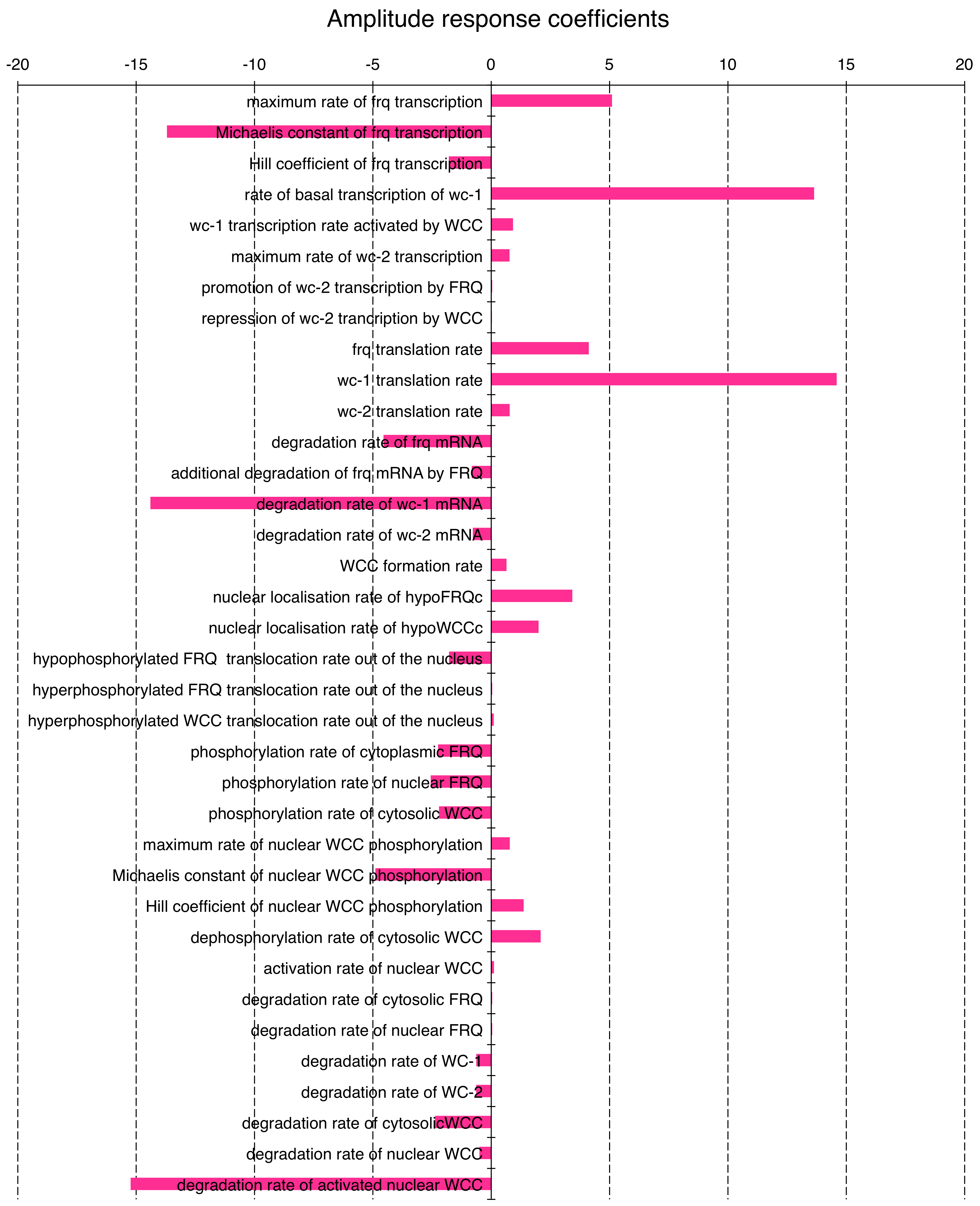
**
