# Supplementary material for: Comprehensive Modelling of the Neurospora Circadian Clock and Its Temperature Compensation
Source: PLoS Comput Biol. 2012 Mar 29;8(3):e1002437. doi: 10.1371/journal.pcbi.1002437 (PMC3320131; doi:10.1371/journal.pcbi.1002437)
Supplement: Table S1 — Kinetic equations used in the model. The rate of reaction i is noted v_i. (DOC) [file pcbi.1002437.s004.doc]

**Table S1: Kinetic equations used in the model**

The rate of reaction *i* is noted *v_i.*

*v_1*=

*v_2*=

*v_3*=

*v_4*=

*v_5*=

*v_6*=

*v_7*=$k\_07\times\text{[}wc-2\text{ mRNA}]$

*v_8*=$k\_08\times[vvd\text{ mRNA]}$

*v_9*=$\text{[frq}\_\text{mRNA]}\times(k\_09+[\text{hypoFRQc}]\times k\_09a)$

*v_10*=$\text{ }k\_10\times\text{[}wc-1\text{ mRNA]}$

*v_11*=$k\_11\times\text{[}wc-2\text{ mRNA}]$

*v_12*=$k\_12\times[vvd\text{ mRNA]}$

*v_13*=$k\_13\times\text{[WC1c]}\times\text{[WC2c]}$

*v_14*=$k\_14\times\text{[hypoFRQc]}$

*v_15*=$k\_15\times\text{[hypoWCCc}]$

*v_16*=$k\_16\times[\text{VVDc]}$

*v_17*=$k\_17\times\text{[hypoFRQn}]$

*v_18*=$k\_18\times\text{[hyperFRQn]}$

*v_19*=$k\_19\times\text{[hyperWCCn]}$

*v_20*=$k\_20\times\text{[hypoFRQc}]$

*v_21*=$k\_21\times\text{[hypoFRQn}]$

*v_22*=$k\_22\times\text{[hypoWCCc}]$

*v_23*=$k\_23\times[\text{hypoWCCn}]\times\frac{[\text{hypoFRQn}]^{H\_23}}{K\_02^{H\_23}+[\text{hypoFRQn}]^{H\_23}}$

*v_24*=$k\_24\times\text{[hyperWCCc}]$

*v_25*=$k\_25\times\text{[hypoWCCn}]$

*v_26*=$k\_26\times\text{[hypoWCCn}]$

*v_27*=$k\_27\times\text{[laWCC}]\times\text{[VVDn}]$

*v_28*=$k\_28\times\text{[WVC}]$

*v_29*=$k\_29\times\text{[hyperFRQc}]$

*v_30*=$k\_30\times\text{[hyperFRQn]}$

*v_31*=$k\_31\times\text{[WC1c}]$

*v_32*=$k\_32\times\text{[WC2c}]$

*v_33*=$k\_33\times\text{[hyperWCCc}]$

*v_34*=$k\_34\times\text{[hyperWCCn}]$

*v_35*=$k\_35\times\text{[aWCC]}$

*v_36*=$k\_36\times\text{[laWCC]}$

*v_37*=$k\_37\times\text{[VVDc}]$

*v_38*=$k\_38\times\text{[VVDn}]$

*v_39*=$k\_39\times\text{[WVC}]$
