# Supplementary material for: Comprehensive Modelling of the Neurospora Circadian Clock and Its Temperature Compensation
Source: PLoS Comput Biol. 2012 Mar 29;8(3):e1002437. doi: 10.1371/journal.pcbi.1002437 (PMC3320131; doi:10.1371/journal.pcbi.1002437)
Supplement: Table S2 — List and values of model parameters. (DOC) [file pcbi.1002437.s005.doc]

**Table S2: List and values of model parameters**

| ID | Name | Description | Value |
| --- | --- | --- | --- |
| *k_01* | *k_frq* | maximum rate of *frq* transcription | 7.3 |
| *K_01* | *K_frq* | Michaelis constant of *frq* transcription | 0.1 |
| *H_01* | *H_frq* | Hill coefficient of *frq* transcription | 4 |
| *k_01a* | *kl_frq* | light induced *frq* transcription | 320 |
| *k_02* | *k_wc1* | rate of basal transcription of *wc-1* | 1.19 |
| *k_02a01* | *ka_wc1* | *wc-1* transcription rate activated by WCC | 1.2 |
| *k_02a02* | *kl_wc1* | light induced *wc-1* transcription | 90 |
| *k_03* | *k_wc2* | maximum rate of *wc-2* transcription | 1.6 |
| *k_03a* | *ka_wc2* | promotion of *wc-2* transcription by FRQ | 0.03 |
| *k_03i* | *ki_wc2* | repression of *wc-2* transcription by WCC | 0.03 |
| *k_04* | *kl_vvd* | light induced *vvd* transcription | 800 |
| *k_05* | *k_FRQ* | *frq* translation rate | 0.19 |
| *k_06* | *k_WC1* | *wc-1* translation rate | 0.226 |
| *k_07* | *k_WC2* | *wc-2* translation rate | 1 |
| *k_08* | *k_VVD* | *vvd* translation rate triggered by light activated WCC | 0.68 |
| *k_09* | *kd_frq* | degradation rate of *frq* mRNA | 2 |
| *k_09a* | *kd_frq_FRQ* | additional degradation of *frq* mRNA by FRQ | 0.356 |
| *k_10* | *kd_wc1* | degradation rate of *wc-1* mRNA | 2.4 |
| *k_11* | *kd_wc2* | degradation rate of *wc-2* mRNA | 2.5 |
| *k_12* | *kd_vvd* | degradation rate of *vvd* mRNA | 6.2 |
| *k_13* | *k_WCC* | WCC formation rate | 0.472 |
| *k_14* | *kin_hypoFRQc* | nuclear localisation rate of hypoFRQc | 0.1 |
| *k_15* | *kin_hypoWCCc* | nuclear localisation rate of hypoWCCc | 0.3 |
| *k_16* | *kin_VVDc* | nuclear localisation rate of VVD | 0.3 |
| *k_17* | *kout_hypoFRQn* | hypophosphorylated FRQ translocation rate out of the nucleus | 0.1 |
| *k_18* | *kout_hyperFRQn* | hyperphosphorylated FRQ translocation rate out of the nucleus | 0.3 |
| *k_19* | *kout_hyperWCCn* | hyperphosphorylated WCC translocation rate out of the nucleus | 0.29 |
| *k_20* | *kp_hypoFRQc* | phosphorylation rate of cytoplasmic FRQ | 0.1 |
| *k_21* | *kp_hypoFRQn* | phosphorylation rate of nuclear FRQ | 0.1 |
| *k_22* | *kp_hypoWCCc* | phosphorylation rate of cytosolic WCC | 0.3 |
| *k_23* | *kp_hypoWCCn* | maximum rate of nuclear WCC phosphorylation | 0.6 |
| *K_23* | *Kp_hypoWCCn* | Michaelis constant of nuclear WCC phosphorylation | 0.475 |
| *H_23* | *Hp_hypoWCCn* | Hill coefficient of nuclear WCC phosphorylation | 12 |
| *k_24* | *kdp_hyperWCCc* | dephosphorylation rate of cytosolic WCC | 0.3 |
| *k_25* | *kact_hypoWCCn* | activation rate of nuclear WCC | 0.15 |
| *k_26* | *klact_hypoWCCn* | activation rate of light activated WCC | 0 |
| *k_27* | *k_WVC* | formation rate of WVC | 20 |
| *k_28* | *kdis_WVC* | disassociation rate of WVC | 1.8 |
| *k_29* | *kd_hyperFRQc* | degradation rate of cytosolic FRQ | 0.27 |
| *k_30* | *kd_hyperFRQn* | degradation rate of nuclear FRQ | 0.27 |
| *k_31* | *kd_WC1* | degradation rate of WC-1 | 0.135 |
| *k_32* | *kd_WC2* | degradation rate of WC-2 | 0.085 |
| *k_33* | *kd_hyperWCCc* | degradation rate of cytosolic WCC | 0.05 |
| *k_34* | *kd_hyperWCCn* | degradation rate of nuclear WCC | 0.05 |
| *k_35* | *kd_aWCC* | degradation rate of activated nuclear WCC | 1.29 |
| *k_36* | *kd_laWCC* | degradation rate of light activated WCC | 6 |
| *k_37* | *kd_VVDc* | degradation rate of cytosolic VVD | 0.24 |
| *k_38* | *kd_VVDn* | degradation rate of nuclear VVD | 0.24 |
| *k_39* | *kd_WVC* | degradation rate of WVC | 0.75 |
