# Supplementary material for: Comprehensive Modelling of the Neurospora Circadian Clock and Its Temperature Compensation
Source: PLoS Comput Biol. 2012 Mar 29;8(3):e1002437. doi: 10.1371/journal.pcbi.1002437 (PMC3320131; doi:10.1371/journal.pcbi.1002437)
Supplement: Table S3 — Parameter sensitivity test for oscillations. For each parameter, the table gives the lower and upper value that conserves frq RNA oscillations, as well as the percentage change with respect to its reference value. (DOC) [file pcbi.1002437.s006.doc]

**Table S3: Parameter sensitivity test for oscillations**

For each parameter, the table gives the lower and upper value that conserves *frq* RNA oscillations, as well as the percentage change with respect to its reference value.

| ID | Parameter name | Reference value | Lowest value generating oscillations | Highest value generating oscillations | Percentage change for lowest value | Percentage change for highest value |
| --- | --- | --- | --- | --- | --- | --- |
| k_10 | *kd_wc1* | 2.4 | 1.206 | 2.471 | -49.75 | 2.96 |
| k_35 | *kd_aWCC* | 1.29 | 0.633 | 1.3266 | -50.93 | 2.84 |
| k_06 | *k_WC1* | 0.226 | 0.218 | 0.455 | -3.54 | 101.33 |
| k_02 | *k_wc1* | 1.19 | 1.148 | 2.47 | -3.53 | 107.56 |
| k_25 | *kact_hypoWCCn* | 0.15 | 0.0545 | 0.301 | -63.67 | 100.67 |
| k_02a01 | *ka_wc1* | 1.2 | 0.68 | 18.1 | -43.33 | 1408.33 |
| k_01 | *k_frq* | 7.3 | 6.5 | 125 | -10.96 | 1612.33 |
| k_05 | *k_FRQ* | 0.19 | 0.168 | 3.3 | -11.58 | 1636.84 |
| k_09 | *kd_frq* | 2 | 0 | 2.202 | -100.00 | 10.10 |
| k_21 | *kp_hypoFRQn* | 0.1 | 0 | 0.1146 | -100.00 | 14.60 |
| k_20 | *kp_hypoFRQc* | 0.1 | 0 | 0.1163 | -100.00 | 16.30 |
| k_33 | *kd_hyperWCCc* | 0.05 | 0 | 0.0601 | -100.00 | 20.20 |
| k_22 | *kp_hypoWCCc* | 0.3 | 0 | 0.3615 | -100.00 | 20.50 |
| k_17 | *kout_hypoFRQn* | 0.1 | 0 | 0.1216 | -100.00 | 21.60 |
| k_11 | *kd_wc2* | 2.5 | 0 | 3.71 | -100.00 | 48.40 |
| k_09a | *kd_frq_FRQ* | 0.356 | 0 | 0.564 | -100.00 | 58.43 |
| k_32 | *kd_WC2* | 0.085 | 0 | 0.1405 | -100.00 | 65.29 |
| k_31 | *kd_WC1* | 0.135 | 0 | 0.224 | -100.00 | 65.93 |
| k_34 | *kd_hyperWCCn* | 0.05 | 0 | 0.123 | -100.00 | 146.00 |
| k_03i | *ki_wc2* | 0.03 | 0 | 0.86 | -100.00 | 2766.67 |
| k_14 | *kin_hypoFRQc* | 0.1 | 0.09 | ∞ | -10.00 | ∞ |
| k_15 | *kin_hypoWCCc* | 0.3 | 0.245 | ∞ | -18.33 | ∞ |
| k_24 | *kdp_hyperWCCc* | 0.3 | 0.243 | ∞ | -19.00 | ∞ |
| k_07 | *k_WC2* | 1 | 0.674 | ∞ | -32.60 | ∞ |
| k_03 | *k_wc2* | 1.6 | 1.075 | ∞ | -32.81 | ∞ |
| k_13 | *k_WCC* | 0.472 | 0.285 | ∞ | -39.62 | ∞ |
| k_23 | *kp_hypoWCCn* | 0.6 | 0.346 | ∞ | -42.33 | ∞ |
| k_19 | *kout_hyperWCCn* | 0.29 | 0.062 | ∞ | -78.62 | ∞ |
| k_03a | *ka_wc2* | 0.03 | 0 | ∞ | -100.00 | ∞ |
| k_18 | *kout_hyperFRQn* | 0.3 | 0 | ∞ | -100.00 | ∞ |
| k_29 | *kd_hyperFRQc* | 0.27 | 0 | ∞ | -100.00 | ∞ |
| k_30 | *kd_hyperFRQn* | 0.27 | 0 | ∞ | -100.00 | ∞ |

Parameters are sorted into four categories: (1) parameters constrained to a fairly small range of values, (2) parameters that can decrease to zero, (3) parameters that can increase to infinity, and (4) parameters that can take any value without losing oscillations. The average absolute percentage difference is used to rank the parameters according to their sensitivity.
