# Supplementary material for: Comprehensive Modelling of the Neurospora Circadian Clock and Its Temperature Compensation
Source: PLoS Comput Biol. 2012 Mar 29;8(3):e1002437. doi: 10.1371/journal.pcbi.1002437 (PMC3320131; doi:10.1371/journal.pcbi.1002437)
Supplement: Table S4 — Parameter sensitivity test for period. For each parameter, the table gives the lower and upper value for which a period of 21.6±0.6 hours is obtained for frq RNA oscillations, as well as the percentage change with respect to its reference value. (DOC) [file pcbi.1002437.s007.doc]

**Table S4: Parameter sensitivity test for period**

For each parameter, the table gives the lower and upper value for which a period of 21.6 ± 0.6 hours is obtained for *frq* RNA oscillations, as well as the percentage change with respect to its reference value.

| ID | Parameter name | Reference value | Value for 21 h period | Value for 22.2 h period | Percentage change for 21 h period | Percentage change for 22.2 h period |
| --- | --- | --- | --- | --- | --- | --- |
| k_35 | *kd_aWCC* | 1.29 | 1.232 | arrhythmic | -4.50 | arrhythmic |
| k_14 | *kin_hypoFRQc* | 0.1 | 0.112 | arrhythmic | 12.00 | arrhythmic |
| k_09 | *kd_frq* | 2 | 1.595 | arrhythmic | -20.25 | arrhythmic |
| k_11 | *kd_wc2* | 2.5 | 0.5 | arrhythmic | -80.00 | arrhythmic |
| k_17 | *kout_hypoFRQn* | 0.1 | arrhythmic | 0.006 | arrhythmic | -94.00 |
| k_02a01 | *ka_wc1* | 1.2 | 2.34 | arrhythmic | 95.00 | arrhythmic |
| k_31 | *kd_WC1* | 0.135 | 0 | arrhythmic | -100.00 | arrhythmic |
| k_32 | *kd_WC2* | 0.085 | 0 | arrhythmic | -100.00 | arrhythmic |
| k_23 | *kp_hypoWCCn* | 0.6 | 1.648 | arrhythmic | 174.67 | arrhythmic |
| k_07 | *k_WC2* | 1 | 6.2 | arrhythmic | 520.00 | arrhythmic |
| k_13 | *k_WCC* | 0.472 | 3.35 | arrhythmic | 609.75 | arrhythmic |
| k_03 | *k_wc2* | 1.6 | 28 | arrhythmic | 1650.00 | arrhythmic |
| k_03i | *ki_wc2* | 0.03 | n/a | arrhythmic | n/a | arrhythmic |
| k_09a | *kd_frq_FRQ* | 0.356 | n/a | arrhythmic | n/a | arrhythmic |
| k_20 | *kp_hypoFRQc* | 0.1 | n/a | arrhythmic | n/a | arrhythmic |
| k_21 | *kp_hypoFRQn* | 0.1 | n/a | arrhythmic | n/a | arrhythmic |
| k_02 | *k_wc1* | 1.19 | 1.24 | 1.152 | 4.20 | -3.19 |
| k_06 | *k_WC1* | 0.226 | 0.2352 | 0.2184 | 4.07 | -3.36 |
| k_10 | *kd_wc1* | 2.4 | 2.29 | 2.47 | -4.58 | 2.92 |
| k_05 | *k_FRQ* | 0.19 | 0.21 | 0.17 | 10.53 | -10.53 |
| k_01 | *k_frq* | 7.3 | 8.3 | 6.63 | 13.70 | -9.18 |
| k_25 | *kact_hypoWCCn* | 0.15 | 0.17 | 0.135 | 13.33 | -10.00 |
| k_15 | *kin_hypoWCCc* | 0.3 | 0.36 | 0.256 | 20.00 | -14.67 |
| k_24 | *kdp_hyperWCCc* | 0.3 | 0.36 | 0.255 | 20.00 | -15.00 |
| k_33 | *kd_hyperWCCc* | 0.05 | 0.04 | 0.06 | -20.00 | 20.00 |
| k_22 | *kp_hypoWCCc* | 0.3 | 0.24 | 0.36 | -20.00 | 20.00 |
| k_19 | *kout_hyperWCCn* | 0.29 | 0.45 | 0.21 | 55.17 | -27.59 |
| k_34 | *kd_hyperWCCn* | 0.05 | 0.015 | 0.11 | -70.00 | 120.00 |
| k_03a | *ka_wc2* | 0.03 | 20 | n/a | 66566.67 | n/a |
| k_18 | *kout_hyperFRQn* | 0.3 | n/a | n/a | n/a | n/a |
| k_29 | *kd_hyperFRQc* | 0.27 | n/a | n/a | n/a | n/a |
| k_30 | *kd_hyperFRQn* | 0.27 | n/a | n/a | n/a | n/a |

Parameters are sorted into different categories: (1) the clock becomes arrhythmic before the target value of the period can be reached, (2) the target period values are reached for a fixed change in the parameter value, (3) the oscillations are maintained but no parameter value is able to achieve the desired period length (n/a).
