# Supplementary material for: Comprehensive Modelling of the Neurospora Circadian Clock and Its Temperature Compensation
Source: PLoS Comput Biol. 2012 Mar 29;8(3):e1002437. doi: 10.1371/journal.pcbi.1002437 (PMC3320131; doi:10.1371/journal.pcbi.1002437)
Supplement: Table S5 — Parameter sensitivity test for amplitude. For each parameter, the table gives the lower and upper value for which the amplitude of frq RNA of oscillations is changed by ±5%, as well as the percentage change with respect to its reference value. (DOC) [file pcbi.1002437.s008.doc]

**Table S5: Parameter sensitivity test for amplitude**

For each parameter, the table gives the lower and upper value for which the amplitude of *frq* RNA of oscillations is changed by ± 5 %, as well as the percentage change with respect to its reference value.

| ID | Parameter name | Reference value | Value for 5 % decrease of amplitude (0.741) | Value for 5 % increase of amplitude (0.819) | Percentage change for 5 % decrease of amplitude (0.741) | Percentage change for 5 % increase of amplitude (0.819) |
| --- | --- | --- | --- | --- | --- | --- |
| k_35 | *kd_aWCC* | 1.29 | 1.294 | 1.2856 | 0.31 | -0.34 |
| k_10 | *kd_wc1* | 2.4 | 2.4076 | 2.3916 | 0.32 | -0.35 |
| k_06 | *k_WC1* | 0.226 | 0.22529 | 0.2268 | -0.31 | 0.35 |
| k_02 | *k_wc1* | 1.19 | 1.186 | 1.1945 | -0.34 | 0.38 |
| k_01 | *k_frq* | 7.3 | 7.235 | 7.372 | -0.89 | 0.99 |
| k_09 | *kd_frq* | 2 | 2.02 | 1.978 | 1.00 | -1.10 |
| k_05 | *k_FRQ* | 0.19 | 0.18795 | 0.19242 | -1.08 | 1.27 |
| k_14 | *kin_hypoFRQc* | 0.1 | 0.0987 | 0.1015 | -1.30 | 1.50 |
| k_21 | *kp_hypoFRQn* | 0.1 | 0.10173 | 0.0978 | 1.73 | -2.20 |
| k_33 | *kd_hyperWCCc* | 0.05 | 0.05095 | 0.04898 | 1.90 | -2.04 |
| k_20 | *kp_hypoFRQc* | 0.1 | 0.10196 | 0.0977 | 1.96 | -2.30 |
| k_22 | *kp_hypoWCCc* | 0.3 | 0.3063 | 0.2932 | 2.10 | -2.27 |
| k_24 | *kdp_hyperWCCc* | 0.3 | 0.293432 | 0.3074 | -2.19 | 2.47 |
| k_15 | *kin_hypoWCCc* | 0.3 | 0.293043 | 0.3081 | -2.32 | 2.70 |
| k_17 | *kout_hypoFRQn* | 0.1 | 0.1025 | 0.09724 | 2.50 | -2.76 |
| k_02a01 | *ka_wc1* | 1.2 | 1.141 | 1.265 | -4.92 | 5.42 |
| k_09a | *kd_frq_FRQ* | 0.356 | 0.376 | 0.3344 | 5.62 | -6.07 |
| k_11 | *kd_wc2* | 2.5 | 2.64 | 2.34 | 5.60 | -6.40 |
| k_07 | *k_WC2* | 1 | 0.946 | 1.068 | -5.40 | 6.80 |
| k_23 | *kp_hypoWCCn* | 0.6 | 0.566 | 0.64 | -5.67 | 6.67 |
| k_03 | *k_wc2* | 1.6 | 1.512 | 1.712 | -5.50 | 7.00 |
| k_32 | *kd_WC2* | 0.085 | 0.0908 | 0.0784 | 6.82 | -7.76 |
| k_31 | *kd_WC1* | 0.135 | 0.1445 | 0.1245 | 7.04 | -7.78 |
| k_13 | *k_WCC* | 0.472 | 0.4408 | 0.512 | -6.61 | 8.47 |
| k_34 | *kd_hyperWCCn* | 0.05 | 0.0547 | 0.0453 | 9.40 | -9.40 |
| k_25 | *kact_hypoWCCn* | 0.15 | 0.1212 | n/a | -19.20 | n/a |
| k_19 | *kout_hyperWCCn* | 0.29 | 0.21725 | n/a | -25.09 | n/a |
| k_03i | *ki_wc2* | 0.03 | 0.13 | n/a | 333.33 | n/a |
| k_03a | *ka_wc2* | 0.03 | n/a | 0.326 | n/a | 986.67 |
| k_18 | *kout_hyperFRQn* | 0.3 | n/a | n/a | n/a | n/a |
| k_29 | *kd_hyperFRQc* | 0.27 | n/a | n/a | n/a | n/a |
| k_30 | *kd_hyperFRQn* | 0.27 | n/a | n/a | n/a | n/a |

Parameters are sorted into two categories: (1) the target amplitude values are reached for a fixed change in the parameter value, (2) no parameter value is able to achieve the desired amplitude (n/a).
